# Unintended effects of statins from observational studies in the general population: systematic review and meta-analysis

**DOI:** 10.1186/1741-7015-12-51

**Published:** 2014-03-22

**Authors:** Ana Filipa Macedo, Fiona Claire Taylor, Juan P Casas, Alma Adler, David Prieto-Merino, Shah Ebrahim

**Affiliations:** 1 Cochrane Heart Group, London School of Hygiene and Tropical Medicine, London, UK; 2 Faculty of Health Sciences, University of Beira Interior, Covilhã, Portugal; 3 Department of Non-communicable Disease Epidemiology, London School of Hygiene and Tropical Medicine, London, UK; 4 Institute of Cardiovascular Science, University College London, London, UK; 5 Department of Medical Statistics, London School of Hygiene and Tropical Medicine, London, UK

**Keywords:** Statins, Unintended effects, Systematic review, Meta-analysis, Observational studies

## Abstract

**Background:**

Efficacy of statins has been extensively studied, with much less information reported on their unintended effects. Evidence from randomized controlled trials (RCTs) on unintended effects is often insufficient to support hypotheses generated from observational studies. We aimed to systematically assess unintended effects of statins from observational studies in general populations with comparison of the findings where possible with those derived from randomized trials.

**Methods:**

Medline (1998 to January 2012, week 3) and Embase (1998 to 2012, week 6) were searched using the standard BMJ Cohort studies filter. The search was supplemented with reference lists of all identified studies and contact with experts in the field. We included prospective studies with a sample size larger than 1,000 participants, case control (of any size) and routine health service linkage studies of over at least one year duration. Studies in subgroups of patients or follow-up of patient case series were excluded, as well as hospital-based cohort studies.

**Results:**

Ninety studies were identified, reporting on 48 different unintended effects. Statins were associated with lower risks of dementia and cognitive impairment, venous thrombo-embolism, fractures and pneumonia, but these findings were attenuated in analyses restricted to higher quality studies (respectively: OR 0.74 (95% CI 0.62 to 0.87); OR 0.92 (95% CI 0.81 to 1.03); OR 0.97 (95% CI 0.88 to 1.05); OR 0.92 (95% CI 0.83 to 1.02)); and marked heterogeneity of effects across studies remained. Statin use was not related to any increased risk of depression, common eye diseases, renal disorders or arthritis. There was evidence of an increased risk of myopathy, raised liver enzymes and diabetes (respectively: OR 2.63 (95% CI 1.50 to 4.61); OR 1.54 (95% CI 1.47 to 1.62); OR 1.31 (95% CI 0.99 to 1.73)).

**Conclusions:**

Our systematic review and meta-analyses indicate that high quality observational data can provide relevant evidence on unintended effects of statins to add to the evidence from RCTs. The absolute excess risk of the observed harmful unintended effects of statins is very small compared to the beneficial effects of statins on major cardiovascular events.

## Background

Randomized controlled trials (RCTs) of statins have demonstrated their efficacy in preventing cardiovascular diseases (CVD) but much less information has been reported on their unintended effects [1-6]. In RCTs not all harmful effects can be easily anticipated, but even if measured, their reporting is inadequate [7]. Under-reporting of unintended effects may affect the interpretation of the net clinical benefit, particularly among people at low cardiovascular risk.

The Cholesterol Treatment Trialists’ (CTT) collaboration, an individual patient data overview of statin trials, has provided strong evidence of benefit across all risk categories from secondary prevention to primary prevention [8-11]. The CTT have confirmed an increased risk of myopathies (including rhabdomyolysis) and found no evidence of any increased risk of cancers [10,11].

Two recent meta-analyses of randomized trials have suggested that statins might be associated with a 9% increased relative risk of type 2 diabetes [12,13]. This led to new safety alerts from both the USA Food and Drugs Administration and the UK Medicines and Health-products Regulatory Agency (MHRA) [14,15]. Statin-induced liver dysfunction also occurs, but its incidence in the general population, in contrast to trials, is not well defined.

Since the start of widespread use of statins in clinical practice, numerous observational studies in North America and Europe have provided contradictory results on the effect of statins on a wide range of unintended effects [16-21]. The lack of coherence is not surprising given the inherent limitations in observational study designs, but is a source of considerable anxiety for the general public. Unfortunately, more robust evidence from RCTs on such unintended effects is often insufficient to support or refute hypotheses generated from observational studies, either because the relevant data were not collected or not reported [22-27].

Although potential biases and confounding have to be considered, population-based observational studies are more likely to include a broad representation of the population typically treated with statins. For these reasons, our objective was to systematically assess unintended effects of statins (with the exception of cancers) from observational studies in the general population.

## Methods

### Criteria for inclusion of studies

This review focused on observational studies of any unintended effects in general populations taking statins, compared with non-users. We included prospective studies with sample sizes larger than 1,000 participants, case control studies (of any size) and routine health service linkage studies of over at least one-year duration. Studies in subgroups of patients (for example, patients with familial hypercholesterolemia) or follow-up of patient case series were excluded, as well as hospital-based cohort studies (that is, cohort studies where the participants were selected among patients admitted to a certain hospital). Studies assessing lipid lowering drugs were included only if separate data on statins were presented, or if the study reported that statins accounted for the majority of the lipid lowering drugs used.

For purposes of this review, an unintended effect was any observational outcome reported (with the exception of cancers as there are no longer any serious concerns about excess cancer risk associated with statin use [11,28]), either favorable or untoward. Efficacy endpoints of reduction in low-density lipoprotein (LDL) cholesterol and total cholesterol were not considered but observational assessments of cardiovascular endpoints were made to test the validity of the approach.

### Search strategy

We searched Medline (1998 to January 2012, week 3) and Embase (1998 to 2012, week 6), using the standard BMJ Cohort studies filter [29]. The search was limited to human studies, with no language restrictions (see Additional file 1: Text S1 for search strategy). Our search strategy aimed to detect observational studies that were concerned with statins used for treatment of cardiovascular diseases. We did not attempt to search with terms related to unintended consequences as specific difficulties arise when ‘unintended effect’ terms are added to a search strategy, because they are poorly reported, inadequately indexed, inconsistently described and can be new or unexpected at the time of searching [30]. Since no search filter seemed to have high sensitivity and specificity in both Medline and Embase databases [30], we supplemented our search with reference lists of all identified studies (including reviews), and contacted experts in the field asking for additional relevant articles.

### Study selection and data extraction

One author (AFM) screened all titles and abstracts for studies that could potentially meet the inclusion criteria (Additional file 1: Text S2). Full articles were retrieved for further detailed assessment by the same reviewer, who then extracted onto a predesigned form information on study design, sample size, study location and duration, drug use and definition of exposure to statins, characteristics of participants, relevant outcomes and effect estimates, and degree of adjustment.

Two authors (FT and AA) checked a random sample of 10% of the titles and the numbers extracted. This approach aimed to assess the magnitude of the discrepancies, which might then lead to a decision to double-check all articles. Since no discrepancies were identified, no further duplicate checking was conducted. We also contacted 71 authors to request additional information, ask for unpublished studies or clarify any uncertainty; 38 of them provided additional information.

### Assessment of risk of bias

The risk of bias in observational studies was assessed using Newcastle-Ottawa Quality Assessment Scale (NOS) (Additional file 1: Text S3) according to the procedures recommended in the Cochrane Handbook of Systematic Reviews [31]. One author (AFM) assessed the methodological quality of all the selected studies.

The NOS includes a ‘star system’ in which a study is judged on three domains: representativeness of study group selection (four items); comparability of groups (two items); and ascertainment of either the exposure or outcome (three items). When any item of NOS (for example, adequacy of follow-up period) was not reported, a zero score was allocated. Studies score one star for each area addressed, with scores between 0 and 9 (the highest level of quality).

### Data analysis

All meta-analyses used random effects models. For binary outcomes, we assumed interchangeability of odds ratio (OR), relative risk (RR) or hazard ratio (HR). Since the majority of unintended effects of statins are fairly rare, these measures will be similar [32]. Sensitivity analyses were carried out according to study design to explore the influence of different measures on the overall estimate of effect. The natural logarithms of effect estimates with 95% confidence intervals were pooled across studies using DerSimonian and Laird random-effects methods. If more than one effect estimate was reported, the one derived from the most adjusted model and with the longest follow-up was used. Where estimates for a study were provided for men and women, these were pooled into a single estimate.

For continuous outcomes reported in different units, we used the adjusted mean change from baseline to follow-up, for exposed and unexposed, to obtain the standardized mean difference (SMD). Analysis was conducted using the metan command in Stata software (version 12.0 by Stata Press, 4905 Lakeway Drive, College Station, Texas).

### Heterogeneity

Because this review included only observational studies, a greater level of heterogeneity was expected. We used I^2^ statistic to assess between-study heterogeneity. Sensitivity analyses were undertaken to explore the influence of different variables on the overall estimate of effect and as potential sources of heterogeneity: study design (cohort versus case-control studies), quality of the study (higher quality for NOS ≥8 vs. NOS <8) and sample size (within three categories: ≤100,000, 100,000 to 1 million, >1 million).

We undertook this systematic review and meta-analysis according to PRISMA (Preferred Reporting Items for Systematic Reviews and Meta-Analyses) and MOOSE (Meta-analysis of Observational Studies in Epidemiology) guidelines during all stages of design, implementation and reporting (see Additional file 1: Text S4 and Additional file 1: Text S5 checklists). No original protocol for the review was produced.

## Results

**Figure 1:**
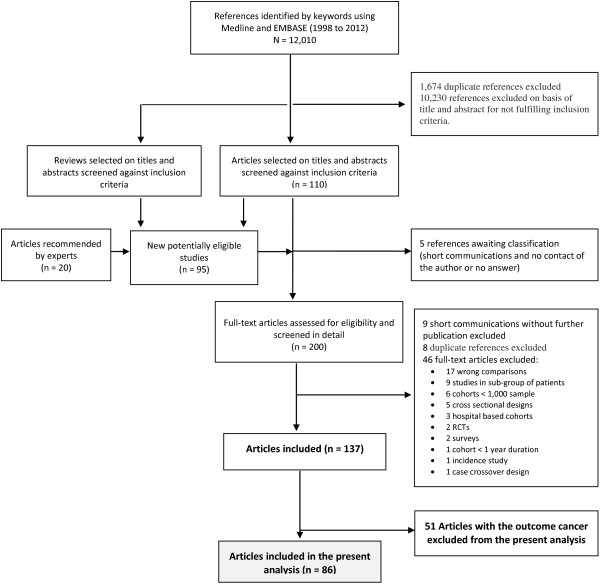
**Flow chart for identifying eligible studies.** The search strategy yielded 12,010 publications, of which 1,674 were duplicates and 10,230 references were not eligible based on abstract and title review. We systematically searched the reference lists of the remaining 110 eligible articles and of 33 reviews, and contacted experts. Ninety-five additional studies were identified that were not captured with our initial search strategy. Of this total, five references of short communications await classification because we were unable to contact the author or received no answer regarding a possible publication. Full papers were obtained for 200 references. From these, 63 were excluded for not fulfilling inclusion criteria. Of the identified 137 references, 51 assessed statin risk of cancer and were excluded from the present analysis; we focused on the remaining 86 references.

Figure 1 summarizes the study selection process (see Additional file 1: Text S2 for studies references). Additional file 1: Table S1 summarizes the characteristics of the 86 articles included, regarding study design, dataset, study population, sample size, follow-up duration and statin type. The analysis comprised 90 studies, 43 cohort studies and 47 case-control studies [17-20,33-114]. Only 6% of the studies were conducted outside Europe (51%) or North America (43%). The mean age of participants ranged from 47.0 to 81.2 years and among the 21 studies that reported data on ethnicity, the most commonly investigated ethnic group was Caucasians (75%).

Forty-eight potential unintended effects of statins were identified, that is, described in at least one study. Definition of exposure to statins was assessed in numerous ways and included different types of statins. In 58 studies, exposure to statins was defined by at least one prescription or self-reported use during the study period.

**Figure 2:**
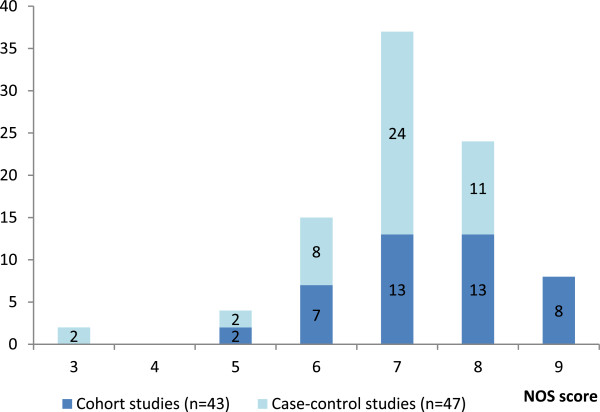
Newcastle-Ottawa Quality Assessment: number of studies by number of “stars” assigned, according to study design.

A summary of the rating of risk of bias according to NOS is presented in Figure 2. The overall quality of the studies was high (mean score 7.1 out of a total possible of nine). All but six studies received six to nine “stars” in the NOS. The mean value of “assigned stars” was 7.4 (range 5 to 9) in the cohort studies and 6.8 (range 3 to 8) in the case control studies.

### Meta-analysis

**Figure 3:**
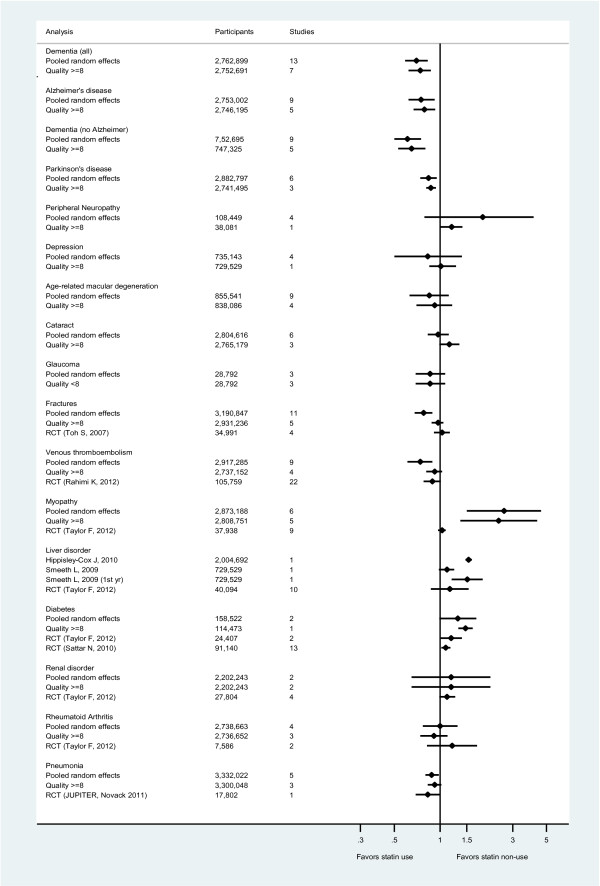
Summary of results from random-effects meta-analyses for each outcome and estimates from Randomized Controlled Trials.

The summary effect estimates of each study were pooled to give a total estimate of risk for each outcome (Figure 3). Effect sizes from meta-analyses of RCTs or single large RCTs were also plotted when available for comparative purposes.

### Neurologic and psychological effects

Thirteen studies with 2,762,899 participants examined the association between use of statins and incidence of any combination of the dichotomous outcomes: dementia, cognitive impairment without dementia (CIND) or Alzheimer’s disease (AD). An odds ratio of 0.70 (95% CI 0.59 to 0.83) was observed for those participants exposed to statins; but the higher quality studies showed an attenuated effect (OR 0.74, 95% CI 0.62 to 0.87) compared with the lower quality studies (OR 0.53, 95% CI 0.37 to 0.92) (Additional file 1: Figure S1-A). Marked between-study heterogeneity as indicated by high I^2^ values was observed which was not explained by stratifying by study quality. Similar association of statin use was observed when the analysis was restricted to incident Alzheimer’s disease (OR = 0.61; 95% CI 0.50 to 0.75), dementia (not Alzheimer’s) (OR = 0.73; 95% CI 0.61 to 0.88) and for incident Parkinson’s disease (OR = 0.84; 95% CI 0.74 to 0.95). One case control study recruited prevalent cases of Parkinson’s disease and observed a stronger risk reduction (OR = 0.37; 95% CI 0.19 to 0.72) [56]. This study was excluded from the pooled analysis because it was responsible for significant heterogeneity between studies (I^2^ = 63.2% vs. I^2^ = 51.8%).

Improved cognitive test scores were observed in two studies: standardized mean difference of 0.18 (95% CI 0.09 to 0.27) [19,101]. In a cohort study of 21 years, statin treatment was related to better performance in episodic memory and psychomotor speed tests (5 to 8% relative differences) [96].

Four studies (108,449 participants) reported on peripheral neuropathy and no strong evidence of increased risk was observed (OR = 1.91; 95% CI 0.89 to 4.41, I^2^ = 73.9%, random effects model) [33,39,48,49]. Studies of low quality (NOS <8) showed a more pronounced association (OR = 2.57; 95% CI 1.04 to 6.32) [33,48,49].

Four studies (735,143 participants) reported on depression and suicidal behavior [17,70,94]. No evidence of increased risk was seen for these endpoints (OR = 0.83; 95% CI 0.50 to 1.39) and this result was consistent across sub-groups.

### Eye disorders

The outcomes comprised age-related macular degeneration (AMD), cataract and glaucoma. No strong evidence of association was observed in the pooled analysis using random effects model for any of these outcomes (Figure 3).

### Venous thromboembolism (VTE)

Using a random effects model, a relative risk reduction of VTE, which favored statin treatment by 26%, was observed (OR = 0.74; 95% CI 0.61 to 0.89). There was marked heterogeneity of effects across studies (I^2^ = 84.1%). Analysis restricted to the four studies with higher quality (NOS ≥8), including 2,737,152 participants, showed marked attenuation of the risk reduction (OR = 0.92; 95% CI 0.81 to -1.03).

### Fractures

A relative risk reduction of fractures on statin treatment by 22% was observed (OR = 0.78; 95% CI 0.68 to 0.89, I^2^ = 91.2%) but this was markedly attenuated when analysis was restricted to the five higher quality studies (OR = 0.97; 95% CI 0.88 to 1.05) and in the cohort studies (OR = 0.85; 95% CI 0.72 to 1.00) and in the three largest studies (OR = 0.98; 95% CI 0.95 to 1.02; I^2^ = 0.0%) (Additional file 1: Figure S1-B).

### Myopathy

A markedly increased risk of myopathy was observed (OR = 2.63; 95% CI 1.50 to 4.61, I^2^ = 98.2%), which was consistent in sub-groups by study design, quality and size but the heterogeneity remained. In a single study, statin treatment was related to an average reduction in grip strength (kg) from baseline of 0.24 kg (men) to 0.52 kg (women) [34]. No evidence of any increase in myopathy in primary prevention RCTs was found (RR = 1.03, 95% CI 0.97 to 1.09, I^2^ = 41%) [1]. CTT results demonstrated an increased risk of myopathy conferred by statins, and an observed excess of rhabdomyolysis of 1 (SE 1) per 10,000 in the 21 trials of standard statin regimens versus control (14 vs. 9 cases) [9].

Incidence rates of events according to exposure and outcome definition were examined (Table 1), and in prospective studies ranged from 2.30 to 9.06 per 10,000 person-years, with an excess rate between 2.10 and 5.77 per 10,000 person-years. Two studies only reported data on absolute risk of myopathy (26 and 11 per 10,000 participants), corresponding to an estimated excess risk of 22 and 8 per 10,000 participants, respectively [55,94]. One study presented what the authors called “prevalence rates” of any myopathic event, in diabetic and nondiabetic subjects, with rates of 242 and 268 per 10,000 person-years, respectively [78].

**Table 1:** Incidence rate and excess risk attributable to statins for myopathy, hepatic disorder and diabetes

**Author, year**	**Incidence/10.000 person-years or % (95% CI)**	**Excess risk/10.000 person-years or %**	**Exposure definition**	**Outcome definition**
Myopathy				
**Gaist *et al.*** [50]	2.30	2.10	One prescription and additional 30 days supply	Medical codes for myopathic events and validation by independent blind review of medical records
	(1.20 to 4.40)			
**Hippisley-Cox *et al.*** [55]	0.26%	0.22%	First prescription after January 2002	Medical codes for moderate or serious myopathic events (myopathy or rhabdomyolysis or a CK ≥4X ULN)
	(0.24% to 0.28%)			
**McClure *et al.*** [77]	9.06	5.77	One prescription during the study period	Medical codes for myopathy and CK ≥10X ULN
	(n.r.)			
**Smeeth *et al.*** [94]	0.11%	0.08%	First prescription after January 1995	Medical codes for myositis or myolysis
	(n.r.)			
Hepatic disorder				
**Hippisley-Cox *et al.*** [55]	1.20%	0.50%	First prescription after January 2002	ALT ≥120 IU/l among patients without chronic liver disease
	(1.13% to 1.22%)			
**Smeeth *et al.*** [94]	1.60%	0.30%	First prescription after January 1995	Medical codes for acute liver disease
**First year only**	0.44%	0.16%		
	(n.r.)			
Diabetes				
**Culver *et al.*** [41]	6.25%	2.25%	One prescription at the first screening interview and three-year follow-up	Self-report of a new physician diagnosis of treated diabetes
	(5.71% to 6.84%)			
**Jick *et al.*** [59]	n.a.	n.a.	Two prescriptions one year before index date	Medical code for first diagnosis of diabetes plus more than two prescriptions for a hypoglycaemic agent or three records of diet management

ALT, alanine transaminase; CK, creatine kinase; n.a., not applicable in a nested case control study; n.r., confidence intervals not reported; ULN, upper limit of normal.

### Liver disorder and raised liver enzymes

The definition of hepatic disorders was ascertained by medical codes for “acute liver disease” in the study by Smeeth *et al*. [94], and by an ALT ≥120 IU/l among patients without chronic liver disease in the study by Hippisley-Cox *et al*. [55] Both studies observed an increased risk of liver disorders. Smeeth *et al*. found an increased risk of incident liver disease in the first year after the index date (HR 1.51; 99% CI 1.19 to 1.91), but little or no increased risk after this time (HR 1.11; 99% CI 0.98 to 1.24); and Hippisley-Cox *et al*. found an increased risk of liver enzyme changes (HR = 1.54; 95% CI 1.47 to 1.62). Results were not pooled across the two definitions of liver disorders. Incidence of liver disorders in these two large prospective studies ranged from 44 to 120 per 10,000 participants, corresponding to an estimated excess risk of 16 and 50 per 10,000 participants, respectively (Table 1) [55,94]. In two additional studies, both gallstones and cholecystectomy events were less common in patients treated with statins (Additional file 1: Figure S2).

### Renal disorder

No evidence of increased risk of renal disorder was observed (OR = 1.18; 95% CI 0.65 to 2.14, I^2^ = 99.2%). The observed heterogeneity might be due to different outcome definitions. The study by Hippisley-Cox *et al*. found an increased risk of “acute renal failure” ascertained by medical codes [55]; while the study by Sukhija *et al*. [100] found a protective effect of statins on renal dysfunction. A moderate rise in urinary albumin excretion (UAE) in statin users (+12.1%) compared with non-users (+3.6%), which was more pronounced with higher dose or duration of statin treatment has been reported, but with no change in glomerular filtration rate (GFR) [35].

### Type 2 diabetes

Overall, weak evidence of an increased risk of type 2 diabetes mellitus (T2DM) was observed (OR = 1.31; 95% CI 0.99 to 1.73, I2 = 72.1%). One cohort study (Women’s Health Initiative) of higher quality and larger sample size found stronger evidence of an increased risk of self-reported T2DM (OR = 1.47; 95% CI 1.32 to 1.64) for the groups of women who reported statin use at baseline and three years later [41]. The cumulative incidence of T2DM after three years of statin treatment was 6.25%, corresponding to an excess risk of 2.25% (Table 1). This excess risk corresponds to a number needed to harm (NNH) of 44 patients (95% CI 35 to 60) for an additional case of diabetes over three years of statin treatment. Conversely, a nested case-control study using data from the General Practice Research Database (GPRD) found no association between statin use and T2DM development (OR = 1.10; 95% CI 0.83 to 1.46) [59].

### Rheumatoid arthritis and osteoarthritis

No increased risk of rheumatoid arthritis or osteoarthritis was observed (OR = 1.00; 95% CI 0.77 to 1.30, I^2^ = 75.7%), which was confirmed in the two cohort studies with higher quality and sample size (OR = 1.00; 95% CI 0.93 to 1.08), while the two case control studies reported conflicting results (Additional file 1: Figure S1-C).

One cohort study reported on the progression of osteoarthritis and observed a protective effect of statins (OR = 0.43; 95% CI 0.25 to 0.77) [38]. No association between statin treatment and other outcomes with a possible immune mechanism (psoriasis, multiple sclerosis or systemic lupus erythematosus) was found (Additional file 1: Figure S2).

### Pneumonia

A risk reduction of pneumonia was observed (OR = 0.88; 95% CI 0.80 to 0.98, I^2^ = 61.7%), which was attenuated in analysis restricted to the three cohort studies with higher quality (NOS ≥8) (OR = 0.92; 95% CI 0.83 to 1.02) (Additional file 1: Figure S1-C) [46,64,94]. Smeeth *et al*. [94] and Fleming *et al*. [46] also found no association between statin treatment and infection-related outcomes, such as acute bronchitis, acute respiratory infections, other respiratory tract infections (no pneumonia) or urinary tract infections, (Additional file 1: Figure S2).

### Other possible unintended effects

No evidence of excess risk of other possible unintended effects that have been suggested was found (Additional file 1: Figure S2). Expected risk reductions were observed for stroke and myocardial infarction. Some unexpected risk reductions were found. Current statin use was associated with a reduced risk of chronic obstructive pulmonary disease (COPD) (OR = 0.45; 95% CI 0.25 to 0.80) in a single study of only one-year duration in which the role of smoking was unclear [88]. The other studies reporting protective associations between statin use and low urinary tract symptoms, subarachnoid hemorrhage and intra-cerebral hemorrhage were all small with poor assigned quality (NOS ≤6) [84,99,110].

## Discussion

We found no increased risk of peripheral neuropathy, depression, common eye diseases, renal disorders or arthritis associated with taking statins. Studies of higher quality did not show previously reported protective effects of statins on fractures, venous thrombo-embolism or pneumonia. Lower odds of dementia and cognitive impairment were associated with statin use but were attenuated in higher quality studies indicating that they may not be robust. There was evidence of an increase in myopathy, raised liver enzymes and diabetes. The pooled observational findings were heterogeneous which might reflect differences in study quality, the populations studied and how statin exposure and effects were measured.

Observational studies of effects of drugs are beset with problems of ‘confounding by indication’ which results in people on treatment often having worse outcomes than those not on treatment simply because they are sicker. In the studies reviewed here, investigators attempted to obtain comparable groups by a range of methods including simple matching, controlling for potential confounders and propensity score matching. None of these methods are as efficient as randomization in controlling for selection bias and confounding but for rare outcomes and to study typical patients using drugs in the general population, observational studies provide the only means of making assessments. Moreover, population-based observational studies provide reliable estimates of the incidence of effects experienced in the general population, important in evaluating the public health impact of an intervention, as this usually cannot be done reliably using randomized trial data (because of limited power and differences between trial samples and general populations).

### Comparison of results with randomized trials

For unintended effects that had been reported in RCTs, the effect estimate derived from observational studies was compared with RCT findings to determine the extent to which observational studies were capable of obtaining robust findings. This approach has been used previously [94]; and a recent study found no difference in the risk estimate of adverse effects of interventions derived from meta-analyses of RCTs and meta-analyses of observational studies, suggesting that these may be estimated reliably from observational data [115]. For this comparison we used results from published data from the CTT collaboration, systematic reviews from the Cochrane collaboration or recent systematic reviews on specific unintended effects that superseded either of these sources. Pooled or single RCT estimates are presented below the observational pooled estimates for the higher quality studies and indicate that in the RCT and observational findings were broadly similar (Figure 3), although RCT estimates tended to be more conservative. Consistency between observational and RCT findings suggests that confounding by indication is unlikely to invalidate the observational results, particularly for estimates obtained from large, better quality observational studies.

Evidence from recent systematic reviews and meta-analyses of randomized trials showed no differences in risk of psychological outcomes [116], fractures [117], acute renal failure [118], arthritis [1] or venous thromboembolism [119]. A recent meta-analysis of randomized trials and prospective cohort studies concluded that long-term data may support a beneficial effect of statins in the prevention of dementia [120] and another review of statins and cognitive impairment found no strong evidence of an association with Alzheimer disease or cognitive function [121].

A recently published meta-analysis of observational studies also found a similar reduction in the likelihood of pneumonia associated with statin use [122], in line with randomized findings from the Justification for the Use of Statins in Primary Prevention: an Intervention Trial Evaluating Rosuvastatin (JUPITER) trial [123]. Statin use may be a surrogate marker for better health which may confound the observed association with pneumonia [124].

RCTs in both primary and secondary prevention found an increased risk of T2DM (OR 1.18; 95% CI 1.01 to 1.39 and OR = 1.09; 95% CI 1.02 to 1.17) [1,12]. This association appears to be higher with intensive-dose statin therapy and among elderly subjects [125], and is strongly associated with baseline fasting blood glucose and co-existing CVD risk factors, suggesting that statins raise blood glucose by a small amount, moving people from below to above the diagnostic threshold [126]. In a recent observational cohort study, atorvastatin and simvastatin compared with pravastatin were associated with an increased risk of new onset diabetes [127]. In a new analysis of the JUPITER primary prevention trial, the cardiovascular and mortality benefits of statin therapy exceeded the risk of developing diabetes even in people at high risk of developing diabetes [128]. Although the mechanism underlying the increase in new onset T2DM in patients treated with statins is unknown, it is possible that statins interfere with insulin signaling, leading to hyperinsulinemia, insulin resistance, metabolic syndrome, pre-diabetes and diabetes [129-131].

The apparent protective effect of statin use on cognitive function was linked with ApoE4 genotype in one study among individuals <80 years [68]. Further research is needed to assess the possible interaction of statin use and ApoE genotype on risk of AD or dementia.

Our analysis gave higher estimates than RCTs for myopathy which may reflect differences in the diagnostic criteria used and the populations studied. Myopathy is more likely to occur with higher doses of statins and in situations that raise statin blood levels, including advanced age, frailty, deterioration of renal function and the presence of interacting drugs [132], all common exclusions for RCTs. A blinded, controlled trial among healthy statin-naïve people showed that high-dose atorvastatin for six months had no effect on muscle strength or exercise performance but confirmed that statins increase muscle complaints and increased average creatine kinase, suggesting that statins produce mild muscle injury even among asymptomatic subjects [133]. An observational study using military personnel records found that musculoskeletal conditions, arthropathies, injuries and pain were more common among statin users than among propensity score-matched nonusers [134].

No increased risk of renal disease attributable to statin treatment was found in our review. Recent systematic reviews of trials have demonstrated lower mortality and cardiovascular events in persons with early stages of chronic kidney disease (CKD) on statins, but little or no effect in persons receiving dialysis, and uncertain effects in kidney transplant recipients [135,136].

We found an increased risk of liver disorders, although outcome definition was heterogeneous across studies. With the exception of statin-induced transaminase elevation, large statins trials have not shown an increase in the incidence of liver diseases. In a systematic review of 21 randomized trials, elevated transaminases occurred with an excess rate of 7 per 10,000 person-years but liver disease was rare (0.05 per 10,000 person-years) [137]. This was confirmed in a larger network meta-analysis [138]. Routine monitoring of liver function after starting a statin is no longer recommended [14].

### Limitations and strengths

One major strength of our systematic review is the inclusion of a large number of observational studies in general populations and a wide range of potential unintended effects of statins. Therefore, our analysis is more likely to include a broad representation of the population at-risk, and may reflect better the nature and frequency of unintended effects experienced in clinical practice.

Despite the many positive aspects, observational research is prone to bias and confounding. The results from the studies included in this meta-analysis could be influenced by a “healthy-user” effect and closer monitoring. We explored the robustness of our results across three dimensions: study design, sample size and quality of the studies but heterogeneity of the pooled observational findings could not be explained by them, and may reflect the different populations studied, how the statin exposure was measured or different outcome definitions. It was not possible to assess the consistency of outcome definitions between studies which is an inherent limitation of systematic reviews based on published data. We also recognize the limitation of having a single reviewer assessing all data extraction and validity; although no discrepancies were identified in a random sample of 10% of the titles and the numbers extracted. Publication bias is a particular threat to the validity of meta-analysis of observational studies. Although we contacted authors asking for unpublished studies, publication bias is possible since observational studies with significant outcomes are more likely to be published and, therefore, over-represented in our meta-analysis. The pooled effect sizes may potentially be exaggerated by this bias.

A further limitation is the difficulty in keeping this review up to date given the high level of publication on statins. In recognition of this we conducted a rapid update search of Medline (January 2012 to February 2014, terms: statins, humans, core medical journals, 141 hits) and have incorporated substantive new findings in relevant sections of our discussion.

Some unintended effects (for example, ventricular arrhythmia, intracerebral hemorrhage) were not covered in this meta-analysis as they were not reported in population-based studies, but from case reports and clinical case series which provide weaker evidence of causal association. A recent meta-analysis including RCTs and observational studies (mainly secondary analysis of RCTs and small studies in subgroups of patients with atrial fribrillation or acute ischemic stroke) found no association between statin exposure and intracerebral hemorrhage, consistent across study designs [139].

### Implications for clinical practice

Statins are widely used in clinical practice and their efficacy for secondary prevention of CVD is well founded, but their expanding use in primary prevention in low-risk individuals has to be balanced against the risk of ‘overmedicating’ the general population [140]. This assumes particular importance since findings from a recent cohort of 3.8 million general population patients of UK Clinical Practice Research Datalink (CPRD) showed substantive overuse of statins in low CVD risk and underuse in high CVD risk [141]. Given that several trials are currently evaluating the effects of a polypill on risk factor levels, the use of statins is likely to increase to a much wider section of the population (whether by age or by cut-off on absolute risk) [142]. Thus, accurate evidence on the potential unintended effects of statins is of public health relevance.

The observed unintended effects, most importantly myopathy and elevated liver enzymes, are rare and more likely to occur with higher doses of statins. The possible increased risk of developing T2DM has resulted in surveillance recommendations.

### Implications for future research

Reporting of unintended effects in RCTs is not done consistently and failure to report such findings introduces bias into evaluations of the benefits and harms of treatments. Considerable adverse event data are collected in most RCTs but are not readily available to include in systematic reviews. Future research in this area will need to engage with pharmaceutical companies to obtain relevant data to enable unbiased assessments of the true levels of common unintended effects of statins.

## Conclusion

Our systematic review and meta-analyses indicate that high quality observational data can provide reliable and relevant evidence on unintended effects of statins to add to the evidence from RCTs for health care guidance. Comparisons of the observational findings with RCTs, where possible, showed similar estimates, indicating that our approach is capable of making plausible inferences on unintended effects. The absolute excess risk of the observed harmful unintended effects of the statin class of drugs is very small compared to the beneficial effects of statins on major cardiovascular events.

### Ethical approval

An ethics statement was not required for this work.

## Abbreviations

AD: Alzheimer’s disease; AMD: Age-related macular degeneration; CIND: Cognitive impairment without dementia; COPD: Chronic Obstructive Pulmonary Disease; CTT: The Cholesterol Treatment Trialists’ collaboration; CVD: Cardiovascular diseases; GPRD: General Practice Research Database; HR: Hazard ratio; LDL: Low-density lipoprotein; MHRA: Medicines and Health-products Regulatory Agency; MOOSE: Meta-analysis of Observational Studies in Epidemiology; NOS: Newcastle-Ottawa Quality Assessment Scale; OR: Odds ratio; PRISMA: Preferred Reporting Items for Systematic Reviews and Meta-Analyses; RCT: Randomized controlled trial; RR: Relative risk; SMD: Standardized mean difference; T2DM: Type 2 diabetes mellitus; VTE: Venous thromboembolism.

## Competing interests

Ana Filipa Macedo conducted this work during a sabbatical at London School of Hygiene and Tropical Medicine and was employed by the University of Beira Interior, Portugal. She is now employed by Boehringer-Ingelheim which does not produce any statin or other lipid lowering drug. All other authors have no relevant conflicts of interest to declare.

## Authors’ contributions

SE originated the idea, and prepared the review and controlled the content. AFM assessed the relevance and quality of papers, extracted data, wrote to authors, analyzed data and prepared the manuscript. FT originated the idea, and helped screen the relevance and quality of papers. JP contributed to the interpretation of results and revised the manuscript critically for important intellectual content. AA helped screen the quality of the data extracted. DP contributed to the statistical analysis. All authors read and approved the final manuscript.

## Pre-publication history

The pre-publication history for this paper can be accessed here:

http://www.biomedcentral.com/1741-7015/12/51/prepub

## Supplementary Material

Additional file 1: Figure S1 A Summary of results from primary meta-analyses for each outcome: pooled fixed effects and subgroup analysis by quality of the studies, sample size and study design. **Figure S1 B.** Summary of results from primary meta-analyses for each outcome: pooled fixed effects and subgroup analysis by quality of the studies, sample size and study design (continued). **Figure S1 C.** Summary of results from primary meta-analyses for each outcome: pooled fixed effects and subgroup analysis by quality of the studies, sample size and study design (continued). **Figure S2.** Summary of results from primary meta-analyses for other possible unintended outcomes: pooled random effects. **Table S1.** Characteristics of the studies included. **Text S1.** Search strategy overview. **Text S2.** Study references. **Text S3.** Newcastle-Ottawa Quality Assessment Scale. **Text S4.** PRISMA checklist. **Text S5.** MOOSE checklist. **Figure S3 to Figure S19.** Pooled random effects for each outcome. Click here for file
