# Supplementary material for: Unintended effects of statins from observational studies in the general population: systematic review and meta-analysis
Source: BMC Med. 2014 Mar 22;12:51. doi: 10.1186/1741-7015-12-51 (PMC3998050; doi:10.1186/1741-7015-12-51)
Supplement: Additional file 1: Figure S1 A — Summary of results from primary meta-analyses for each outcome: pooled fixed effects and subgroup analysis by quality of the studies, sample size and study design. Figure S1 B. Summary of results from primary meta-analyses for each outcome: pooled fixed effects and subgroup analysis by quality of the studies, sample size and study design (continued). Figure S1 C. Summary of results from primary meta-analyses for each outcome: pooled fixed effects and subgroup analysis by quality of the studies, sample size and study design (continued). Figure S2. Summary of results from primary meta-analyses for other possible unintended outcomes: pooled random effects. Table S1. Characteristics of the studies included. Text S1. Search strategy overview. Text S2. Study references. Text S3. Newcastle-Ottawa Quality Assessment Scale. Text S4. PRISMA checklist. Text S5. MOOSE checklist. Figure S3 to Figure S19. Pooled random effects for each outcome. [file 1741-7015-12-51-S1.docx]

**Additional files:**

**Figure S1 - A. Summary of results from primary meta-analyses for each outcome: pooled fixed effects and subgroup analysis by quality of the studies, sample size and study design.**

**Figure S1 - B. Summary of results from primary meta-analyses for each outcome: pooled fixed effects and subgroup analysis by quality of the studies, sample size and study design (cont.).**

**Figure S1 - C. Summary of results from primary meta-analyses for each outcome: pooled fixed effects and subgroup analysis by quality of the studies, sample size and study design (cont.).**

**Figure S2. Summary of results from primary meta-analyses for other possible unintended outcomes: pooled random effects.**

**Table S1 - Table 1- Characteristics of the studies included**

**Text S1. Search strategy overview.**

**Text S2. Study references.**

**Text S3. Newcastle-Ottawa Quality Assessment Scale**

**Text S4. PRISMA checklist**

**Text S5. MOOSE checklist**

**Figure S3 to Figure S19 – Pooled random effects for each outcome**

**Figure S1 – A**

**Figure S1 – B**

**Figure S1 – C**

**Figure S2**

**Table S1. Characteristics of the studies included.**

| **Author** | **Year** | **Country** | **Design** | **Dataset** | **Population** | **Sample size** | **Age*** | **Male %** | **White %** | **Study duration (Follow-up)** | **Minimum Exposure** | **Statin type†** |  |  |
| --- | --- | --- | --- | --- | --- | --- | --- | --- | --- | --- | --- | --- | --- | --- |
| Anderson | 2005 | USA | Nested Case-control | Intermountain Health Care Database, IHC | Patients using IHC, from Utah and neighbour states | 1632 | 47.0(13.0) | 43.0 | n.r. | 2000 to 2003  (n.r.) | n.r. | n.r |  |  |
| Ashfield | 2010 | UK | Prospective Cohort | The Hertfordshire Cohort Study, HCS | Participants of HCS, aged 59-73 years | 2987 | 66.1(2.9) | 52.6 | n.r. | 1998 to 2004 (all 7y) | 1 prescription during the study period | n.r |  |  |
| Atthobari | 2006 | The Netherlands | Prospective Cohort | The Prevention of Renal and Vascular ENd-stage Disease, PREVEND | Participants of PREVEND, aged 28-75 years | 3440 | 49.3/57.9 | 41.3/58.5 | n.r. | 1987 until mean follow up 4.2y | 1 prescription the year preceding the follow up assessment | A,P,S |  |  |
| Beate Ritz | 2010 | Denmark | Case-control | Population of Denmark | Population of Denmark, 35 years or older | 11582 | 72.2(10.2) | 58.0 | n.r. | 1995 to 2001/06 (median 2.5/2.3y) | 2 prescriptions before index date | A,C,F,L,P,S |  |  |
| Becker | 2008 | UK | Nested Case-control | General Practice Research Database, GPRD | Primary care patients using GPRD, 40 years or older | 7274 | (60+) 91.3/91.2 | 59.6 | n.r. | January 1, 1994 - December 31, 2005 (n.r.) | 1 prescription 90 days before index date | A,F,P,R,S |  |  |
| Bernick | 2005 | USA | Prospective Cohort | The Cardiovascular Health Study, CHS | Participants of the CHS | 2324 | 72.9(3.6)/74.6(5.3) | 44.5/41.7 | n.r. | 1989/92 to 1999 (10y) | more than 4y of continuous use | A,F,L,P,S |  |  |
| Chan | 2000 | USA | Case-control | 6 Health Organisations in USA | Women members of health organisations | 3675 | 76.7 (8.1)/ 75.9 (7.8) | 0 | n.r. | October 1994 to September 1997 (all 2y) | ≥13 dispensings 2 years before index date | A,F,L,P,S |  |  |
| Clockaerts | 2010 | The Netherlands | Prospective Cohort | The Rotterdam Study, RS | Participants of the RS, 55 years or older | 2919 | 64.1/65.9 | 43.7/48.6 | n.r. | 1990/93 to 1996/99 (n.r.) | Use 120 days and/or a daily intake > 50% of the recommended daily adult dose | n.r. |  |  |
| Corrao | 2004 | Italy | Case-control | Hospital and mortality databases | Population of Italy, 40 years or older | 38081 | 60.6/59.6 | 30.3/28.8 | n.r. | 1997 to 1999 (n.r.) | 1 prescription 1 year before index date | A,C,F,P,S |  |  |
| Cramer | 2008 | USA | Prospective Cohort | The Sacramento Area Latino Study on Aging, SALSA | Mexican Americans from Sacramento, 60 years or older | 1674 | 69.6 (6.2) 70.4 (7.0) | 41.0/42.0 | all Mexicans | 1998/99 to 2003/04 (all 5y) | 1 prescription during the study period | A,C,F,L,P,R,S |  |  |
| Culver | 2012 | USA | RCT + Cohort | Women’s Health Initiative (WHI) Observational Study | Women from WHI study, aged 50-79 years | 114473 | 63.2(7.3) | 0 | 83,7 | 1993/98 to 2005 (range 3 to 9y) | 1 prescription on the first screening interview | A,F,L,P,S |  |  |
| de Vries (a) | 2006 | UK | Nested Case-control | General Practice Research Database, GPRD | Primary care patients using GPRD | 113416 | 72.0/71.1 | 24.0 | n.r. | January 1987 to April 2002 (mean 3.4y) | 1 prescription 180 days before index date | n.r |  |  |
| de Vries (b) | 2006 | UK | Nested Case-control | General Practice Research Database, GPRD | Primary care patients using GPRD, 50 years or older | 263676 | 71.6 | 25.0 | n.r. | January 1987 to April 2002 (mean 4.0y) | 1 prescription 180 days before index date | n.r |  |  |
| Doggen | 2003 | USA | Nested Case-control | The Group Health Cooperative, GHC | Postmenopausal women using GHC, aged 30-89 years | 2427 | n.r. | 0 | n.r. | January 1, 1995 to December 31, 2000 (median 1.2/0.96 y) | 1 prescription before index date with enough medication to last until index date, assuming 80% compliance | n.r |  |  |
| Erichsen | 2011 | Denmark | Retrospective Cohort | Medical databases from Denmark | Population from Denmark | 357419 | (60+) 40.2 | 31.2 | n.r. | January 1, 1996 to December 31, 2008 (n.r.) | 1 prescription 90 days before index date | n.r. |  |  |
| Fleming | 2012 | UK | Retrospective Cohort | The Weekly Returns Service, WRS of Royal College of General Practitioners, RCGP | Patients registered in the WRS of the RCGP | 40000 to 50000 each year | (65+) 58.7/51.6 | 54.7/42.8 | n.r. | 8 winter influenza seasons: 1 July 1998 to 30 June 2006 (n.r.) | 2 prescriptions before and 1 prescription after 31 December in the study year | n.r. |  |  |
| Fujii (a) | 2009 | Japan | Case-control | Gunma Prefectural Cardiovascular Centre, GPCC | Patients from GPCC | 360 | 69.0(13.0) | 62.5 | n.r. | December 2005 to October 2008 (n.r.) | Any use 7 days before index date | A,P,Pit,R,S |  |  |
| Fujii (b) | 2009 | Japan | Case-control | Gunma Prefectural Cardiovascular Centre, GPCC | Patients from GPCC | 438 | 66.7(12.3) | 58.2 | n.r. | December 2005 to October 2008 (n.r.) | Any use 7 days before index date | A,P,Pit,R,S |  |  |
| Gaist (1) | 2001 | UK | Retrospective Cohort | General Practice Research Database, GPRD | Patients using GPRD, aged 40-74years | 64437 | 40 to 74y | 56.5 | n.r. | January 1991 to September 1997 (n.r.) | 1 prescription and additional 30days supply | A,C,F,P,S |  |  |
| Gaist (2) | 2001 | UK | Retrospective Cohort | General Practice Research Database, GPRD | Patients using GPRD, aged 40-74years | 64437 | n.r. | n.r. | n.r. | January 1991 to September 1997 (n.r.) | n.r. | A,C,F,P,S |  |  |
| Gaist | 2002 | Denmark | Nested case-control | Hospital and Pharmacy Registry | Population from denamark | 4299 | n.r. | n.r. | n.r. | January 1, 1994 to December 31, 1982 (median 8y) | 1 prescription 90 days before index date | F,L,P,S |  |  |
| Gao | 2012 | USA | Prospective Cohort | The Nurses’ Health Study,NHS and The Health Professional Follow-up Study, HPFS | Female nurses aged 30-55 years from NHS, male health professionals aged 40-75 years from HPFS | 129066 | mean 58.2 to 62.0 | 29.5 | n.r. | 1994 to 2006 (all 12y) | ≥2 times/wk in 1994 | n.r. |  |  |
| Green | 2006 | USA | Case-control | Multi-Institutional Research in Alzheimer’s Study, MIRAGE | Participants of MIRAGE study | 2361 | 70.0(8.2)/65.2(8.8) | 37.2/39.5 | n.r. | May 1996 to May 2002 (n.r.) | Daily use 6 months before index date | A,C,F,L,P,S |  |  |
| Gulmez | 2009 | Denmark | Case-control | Multiple administrative health-care databases | Population of Denmark | 40154 | (60+) 80.6 | 51.2 | n.r. | August 1995 to July 2006 (n.r.) | 1 prescription before index date | A,C,F,L,P,R,S |  |  |
| Haag | 2009 | The Netherlands | Prospective Cohort | The Rotterdam Study, RS | Participants of the RS | 6992 | 69.4(9.1) | 40,0 | n.r. | 1990/93 to January 2005 (mean 9y) | 1 prescription before or at index date | A,C,F,P,R,S |  |  |
| Hippisley-Cox | 2010 | UK | Prospective Cohort | General Practice Research Database, QResearch | Primary care patients using QResearch | 2004692 | 57.2(11.7)44.4(13.7) | 53.6/48.9 | 95.2/ 95.6 | 1 January 2002 to 30 June 2008 (n.r.) | First prescription after January 2002 | A,F,P,S,R |  |  |
| Huang | 2007 | USA | Case-control | Population from North Carolina | Cases from University of North Carolina Movement Disorder Clinic and the spouses as controls | 236 | 67.9/65.7 | 55.6/46.6 | n.r. | July 2002 and November 2004 (n.r.) | 1 prescription before or at index date | n.r. |  |  |
| Huerta | 2007 | UK | Nested Case-control | General Practice Research Database, GPRD | Primary care patients using GPRD, aged 20-79 years | 103006 | 63,0 | n.r. | n.r. | 1994 to 2000 (n.r) | until the index date or ended in the 30 days before | n.r |  |  |
| Jick | 2000 | UK | Nested Case-control | General Practice Research Database, GPRD | Primary care patients using GPRD, aged 50-89 years | 1364 | (+60) 98.0/97.0 | 40.0/39.0 | n.r. | 1 January 1992 to 1 January 1998 (n.r) | 1 prescription before index date | A,C,F,P,S |  |  |
| Jick | 2004 | UK | Nested case-control | General Practice Research Database, GPRD | Primary care patients using GPRD, aged 30-79 years | 44049 | mean 59.2 | 51.0 | n.r. | 1 January 1991 to 31 March 2002 (n.r.) | 2 prescriptions 1year before index date | A,C,F,P,S |  |  |
| Jick | 2009 | UK | Nested case-control | General Practice Research Database, GPRD | Primary care patients using GPRD, aged 40-89years | 1565 | (60+) 60.7/60.6 | 39.3 | n.r. | 1 January 1992 to 31 December 20012(n.r.) | 2 prescriptions 1year before index date | A,C,F,P,S |  |  |
| Jong | 2011 | The Netherlands | Nested case-control | Netherlands Information Network of General Practice, LINH | Patients using LINH, 40 years or older | 2877 | 63.4/62.8 | 32.5/34.2 | n.r. | 2001 to 2006 (n.r.) | 1 prescription 1 year before index date | A,C,F,P,S |  |  |
| Klein | 2004 | USA | Prospective Cohort | The Beaver Dam Eye Study, BDES | Population of Beaver Dam, Wiscosin, 41-91 years | 2780 | 64.1/63.5 | 58.5/57.5 | n.r. | 1993/95 to 1998/2000 (all 5y) | 1 prescription on the first screening interview | A,F,L,P,S |  |  |
| Klein | 2006 | USA | Prospective Cohort | The Beaver Dam Eye Study, BDES | Population of Beaver Dam, Wiscosin, 43-84 years | 1299 | 63.2 | 46.3 | 99,0 | 1998/2000 to 2003/05 (all 5y) | 1 prescription on the first screening interview | A,F,L,P,S |  |  |
| Klein | 2007 | USA | Prospective Cohort | The Beaver Dam Eye Study, BDES | Population of Beaver Dam, Wiscosin, 53-96 years | 2204 | 67.4/66.8 | 48.2/39.6 | 99,0 | 1998/00 to 2003/05 (all 5y) | 1 prescription on the first screening interview | A,F,L,P,S |  |  |
| Kwong | 2009 | Canada | Retrospective Cohort | Multiple Administrative Healthcare Databases | Patients 65 years or older, who received influenza vaccination at least 2 weeks prior to the start of influenza season | 2240638 | 74.3(5.8) | 45.1 | n.r. | 10 influenza seasons: 1996/97 to 2005/06 (n.r.) | 1 prescription 90 days before the influenza season | A,C,F,L,P,S |  |  |
| LaCroix | 2003 | USA | Prospective Cohort | Women’s Health Initiative (WHI) Observational Study | Postmenopausal women from WHI, aged 50–79 years | 93716 | (+60) 82.1/67.1 | 0 | 82.3/ 83.3 | 1994/1998 to 2001 (median 3.9y) | 1 prescription on the first screening interview | A,F,L,P,S |  |  |
| LaCroix | 2008 | USA | Prospective Cohort | Women’s Health Initiative (WHI) Observational Study | Postmenopausal women from WHI, aged 65–79 years | 25378 | (70+) 48.4 | 0 | 89,0 | 1993 to 1998 (all 3y) | 1 prescription on the first screening interview | A,F,L,P,S |  |  |
| Lacut | 2008 | France | Case-control | Hospital-based case-control study EDITH | Participants from EDITH hospital study | 1354 | 67.9/68.0 | 43.3 | n.r. | 2000 to 2004 (n.r.) | use at admission for more than 1 wk | n.r |  |  |
| Lemaitre | 2002 | USA | Prospective Cohort | The Cardiovascular Health Study, CHS | Participants of the CHS | 1914 | 71.1/72.5 | 31.5/36.6 | 80.1/84.7 | June 1989 to June 1992/1997 | use 2 wk before each annual visit | n.r |  |  |
| Li G | 2004 | USA | Prospective Cohort | The Adult Changes in Though study, ACT | Participants of the ACT study, 65 years or older | 1496 | 75.1(6.1) | 40.2 | 91,2 | 1994/96 to 31 December 2002 (range 7 to 9y) | 2 consecutive dispensings within 6 months | A,L,P,S |  |  |
| Li G | 2010 | USA | Prospective Cohort | The Group Health Cooperative, GHC | Members of GHC, 65 years or older | 3099 | 74.2/75.8 | 50.0/38.0 | 90,0 | Recruit in 1994/96 and in 2000/02 (mean 6.1y) | 3 prescriptions during study period | A,L,P,S |  |  |
| Luijendijk | 2008 | The Netherlands | Prospective Cohort | The Rotterdam Study, RS | Participants of the RS, 61 years or older | 2801 | 71.0(6.3) | 42.0 | n.r. | 1997/99 to 2002/04 (mean 5y) | a prescription 90 days before index date | n.r. |  |  |
| Marcus | 2012 | the Netherlands | Prospective Cohort | The Rotterdam Study, RS | Participants of the RS, 55 years or older | 3902 | 64.3/66.1 | 43.4/41.1 | n.r. | 1991/93 to 2002/06 (mean 9.8y) | a prescription 90 days before index date | A,C,F,P,R,S |  |  |
| McClure | 2007 | USA | Retrospective Cohort | Kaiser Permanente Medical Care Program, KPMCP | Members of KPMCP Colorado, aged 40-89 years | 15033 | 61.2 | 45.0 | n.r. | January 1, 1999 to December 31, 2003 (mean 2.1/2.0y) | 1 prescription during the study period | A,C,F,L,P,R,S |  |  |
| McGwin | 2003 | USA | Nested case-control | Veterans Affairs Health Care System, VAHCS | Male patients using VAHCS, 50 years or older | 6050 | 72.9/73.2 | 100 | 83.5/ 45.6 | 1 January 1997 to 31 December 2001 (n.r.) | 1 prescription 180 days before index date | A,C,F,L,P,S |  |  |
| McGwin | 2004 | USA | Nested case-control | Veterans Affairs Health Care System, VAHCS | Male patients using VAHCS, 50 years or older | 7334 | 69.0 | 100 | 37.8/ 47.4 | 1 January 1997 to 31 December 2001 (n.r.) | 1 prescription 180 days before index date | A,C,F,L,P,S |  |  |
| McGwin | 2005 | USA | Nested case-control | The Cardiovascular Health Study, CHS | Participants from the CHS | 2755 | (+76) 83.6/89.4 | 41.0/40.0 | 91.8/ 82.6 | 1989/90 to 1997/98 (n.r.) | 1 prescription 2wk before one follow up visit | n.r. |  |  |
| Meier | 2000 | UK | Nested Case-control | General Practice Research Database, GPRD | Primary care patients using GPRD | 27319 | (+60) 81.6/81.4 | 25.0 | n.r. | 1980 to September 1998 (n.r.) | 1 prescription 30 days before index date | A,C,F,P,S |  |  |
| Myles | 2009 | UK | Nested case-control | The Health Improvement Network database, THIN | Patients using THIN, 40years or older | 25883 | (60+) 73.4 | 46.0 | n.r. | 1 July 2001 to 1 July 2002 (n.r.) | 1 prescription 30 days before index date | A,F,L,P,R,S |  |  |
| Nash | 2011 | USA | Prospective Cohort | The Beaver Dam Offspring Study, BOSS | Participants from BOSS | 2597 | 49.2(9.9) | 45.4 | n.r. | 2005-2008 (n.r.) | self-reported use | n.r. |  |  |
| Nichols(a) | 2007 | USA | Retrospective Cohort | Kaiser Permanente Medical Care Program, KPMCP | Diabetic members of KPMCP Colorado | 20494 | 60.4/56.7 | 51.8/49.6 | n.r. | 1997 to 2004 (mean 3-3.5y) | 1 prescription prior to event date and 1 day's supply allocated for the last observation date | L,S |  |  |
| Nichols(b) | 2007 | USA | Retrospective Cohort | Kaiser Permanente Medical Care Program, KPMCP | Non diabetic members of KPMCP Colorado | 43956 | 61.0/58.7 | 55.9/42.8 | n.r. | 1997 to 2004 (n.r.) | 1 prescription prior to event date and 1 day's supply allocated for the last observation date | L,S |  |  |
| Owen | 2010 | UK | Nested case-control | DIN-LINK | Primary care patients using DIN-LINK | 17556 | 70.0 | 47.0 | n.r. | 2000 to 2007 (all 5y) | 1 prescription during the study period | n.r. |  |  |
| Ramcharan | 2009 | The Netherlands | Case-control | The Multiple Environmental and Genetic Assessment of risk factors for venous thrombosis, MEGA study | Participants from MEGA study, 18-70 years | 10452 | 49.6/48.3 | 45.8/46.2 | n.r. | March 1999 to September 2004 (n.r.) | self-reported use at index date | A,F,P,S,R |  |  |
| Ray | 2002 | USA | Retrospective Cohort | Tennessee Medicaid program | Patients enrolled in the Medicaid program | 29786 | 61.8/61.9 | 34.6/33.8 | 75.6/77.3 | 1 January 1989 to 31 December 1998 (n.r.) | 2 prescriptions 90 days after the first prescription | A,C,F,L,P,S |  |  |
| Rea | 2005 | USA | Prospective Cohort | The Cardiovascular Health Study, CHS | Participants of the CHS, 65 years or older | 2798 | (+80) 56.7/54.5 | 32.7/40.8 | 90.7/90.3 | 1991 to 1994 (n.r.) | 1 prescription during the study period | A,C,F,L,P,S |  |  |
| Rejnmark | 2004 | Denmark | Case-control | Hospital Patient Register and Civil Registration System | Population of North Jutland, Denmark | 39934 | 78.3 | 29.6 | n.r. | 1 January 1989 to 31 December 2001 (all 5y) | 4 prescriptions during 5 years | A,C,F,L,P,S |  |  |
| Risselada | 2009 | The Netherlands | Nested case-control | PHARMO Record Linkage System | Patients using PHARMO | 11037 | median 58.0 | 33.0 | n.r | January 1, 1998 to December 31, 2006 (n.r.) | use at index date | A,C,F,P,R,S |  |  |
| Rockwood | 2002 | Canada | Nested Case-control | The Canadian Study of Health and Aging study, CSHA | Participants of the CSHA, 65 years or older | 1149 | 81.2(6.3)/76.0(6.5) | 30.0/42.0 | n.r. | 1991/92 to 1996/97 (n.r.) | self-reported use before index date | n.r. |  |  |
| Rockwood | 2007 | Canada | Nested Case-control | The Canadian Study of Health and Aging study, CSHA | Participants of the CSHA, 65 years or older | 1040 | 77.9(6.8)/75.9(6.2) | 39.0/42.0 | n.r. | 1991/92 to 1996/97 (n.r.) | self-reported use at index date | n.r. |  |  |
| Rodriguez | 2009 | UK | Nested case-control | General Practice Research Database, GPRD | Patients using GPRD, aged 40-89 years | 18473 | (60+)79.3/80.3 | 52.9/51.0 | n.r. | 1 January and 31 December 1996 (n.r.) | 1 prescription 30days before index date | n.r. |  |  |
| Schliender | 2001 | UK | Nested case-control | General Practice Research Database, GPRD | Primary care patients using GPRD | 35732 | (+60) 87.5/87.1 | 39.2/38.8 | n.r. | 1987 to 30 September 1998 (mean 6.2y) | ≥30 prescriptions before index date | A,C,F,P,S |  |  |
| Schlienger | 2007 | UK | Nested case-control | General Practice Research Database, GPRD | Patients using GPRD, 30 years or older | 6091 | (+60) 73.7/73.2 | 54.3/54.5 | n.r. | 1 January 1995 to 30 April 2002 (n.r.) | 1 prescription 30days before index date | A,F,L,S |  |  |
| Schoofs | 2004 | The Netherlands | Prospective Cohort | The Rotterdam Study, RS | Participants of the RS, 55 years or older | 3469 | 64.3 (5.6)/ 65.7 (6.7) | 43.0 | n.r. | 1990/1993 to 1997/1999 (mean 6.5y) | 1 prescription between the baseline and follow-up radiograph | A,F,L,P,S |  |  |
| Scranton | 2005 | USA | Retrospective Cohort | Veterans Affairs Health Care System, VAHCS | Patients using VAHCS | 88857 | 65.1(10.4)59.3(14.9) | 95.3 | n.r. | 1 January 1998 to 30 June 2001 (n.r.) | 2 prescription during the study period | A,C,F,L,P,S |  |  |
| Smeeth | 2003 | UK | Nested case-control | General Practice Research Database, GPRD | Primary care patients using GPRD | 30958 | 75.0 | 35.4 | n.r. | 1987 to June 2001 (mean 1.6y) | n.r. | A,C,F,P,S |  |  |
| Smeeth | 2005 | UK | Nested case-control | General Practice Research Database, GPRD | Primary care patients using GPRD, 50 years or older | 104176 | 77.4 | 44.1/44.0 | n.r. | June 1987 to April 2002 (mean 1.2y) | 1 prescription during the study period | A,C,F,P,S |  |  |
| Smeeth | 2009 | UK | Prospective Cohort | The Health Improvement Network database, THIN | Primary care patients using THIN | 729529 | (+60) 60.7/61.9 | 50.7/49.8 | n.r. | 1 January 1995 to December 2006 (median 4.4y) | First prescription after January 1995 | A,C,F,P,S,R |  |  |
| Solomon | 2009 | Finland | Prospective Cohort | The Cardiovascular risk factors, aging and dementia study, CAIDE | Participants of the CAIDE | 1382 | n.r. | 37.8 | n.r. | 1972 to 1998 (all 21y) | n.r. | n.r. |  |  |
| Sørensen | 2009 | Denmark | Case-control | General population of North Jutland | General population of North Jutland | 36926 | (71+) 45.5 | 46.6 | n.r. | 1997 to 2005 (n.r.) | 1 prescription 90 days before index date | n.r |  |  |
| Sørensen | 2010 | Denmark | Case-control | The Danish National Registry of Patients | Population of Denmark | 6116 | 66.2(12.0) | 54.3 | n.r. | 1994/99 to 2008 (n.r.) | 1 prescription before index date | n.r. |  |  |
| St Sauver | 2011 | USA | Retrospective Cohort | The Olmsted County Study of Urinary Symptoms and Health Status Among Men | Population of Olmsted | 1661 | (60+) 27.6/29.3 | 100 | 100 | 1990 to 2007 (median 13.8y) | n.r. | n.r. |  |  |
| Sukhija | 2008 | USA | Retrospective Cohort | Veterans Affairs Health Care System, VAHCS | Patients using VAHCS | 197551 | 65.0(14.0) | 98.0 | n.r. | 1 February 2000 to 28 September 2005 (n.r.) | 1 prescription during the study period | A,C,F,L,P,R,S |  |  |
| Szwast | 2007 | USA | Prospective Cohort | Indianapolis Ibadan Dementia Project and Medicare | African Americans from Indiana,70 years or older | 1141 | 77.3(5.3) | 30.7 | all African Americans | 2001 to 2004 (all 3y) | use at follow up visits | A,C,F,L,P,S |  |  |
| Tan (1) | 2007 | Australia | Prospective Cohort | The Blue Mountains Eye Study, BMES | Participants of the BMES, 49 years or older | 2406 | 64.3 | 42.5 | n.r. | 1992/94 to 2002/04 (all 10y) | use at follow up visits | n.r. |  |  |
| Tan (2) | 2007 | Australia | Prospective Cohort | The Blue Mountains Eye Study, BMES | Participants of the BMES, 49 years or older | 2178 | 67.4/66.8 | 48.2/39.6 | 99,0 | 1992/94 to 2002/04 (all 10y) | use at follow up visits | n.r. |  |  |
| Tsai | 2009 | USA | Retrospective Cohort | The Nurses’ Health Study, NHS | Female nurses aged 30-55 years from NHS | 53611 | mean 64.8 to 66.2 | 0 | n.r. | 1994 to 2000 (n.r.) | self-reported use in follow up questionnaire | n.r. |  |  |
| van Leeuwen | 2003 | The Netherlands | Prospective Cohort | The Rotterdam Study, RS | Participants of the RS, 55 years or older | 4681 | n.r. | n.r. | n.r. | Recruit 1990/93 (mean 6.5y) | use for more than 1 y | n.r. |  |  |
| van Leeuwen | 2004 | 3 Continents | 3 Prospective Cohorts | The RS, BMES and BDES studies | Participants from the RS, BMES and BDES studies | 8649 | 62.7 | 43.1 | n.r. | Duration of each study (mean 5.6y) | exposure of each study | n.r. |  |  |
| Van Staa | 2001 | UK | Nested Case-control | General Practice Research Database, GPRD | Primary care patients using GPRD | 163760 | (+70) 51.7/52.5 | 24.0 | n.r. | June 1987 to July 1999 (mean 3.1/2.4y) | 1 prescription 180 days before index date | A,C,F,P,S |  |  |
| Wahner | 2008 | USA | Case-control | The Parkinson’s Environment Genes study, PEG | Medicare patients from PEG study | 654 | 70.0/69.0 | 53.2/49.1 | 81.4/81.0 | 20001 to 2007 (mean 4.4/3.8y) | self-reported use before index date | A,L,P,R,S |  |  |
| Wang | 2000 | USA | Case-control | Medicaid program | Patients enrolled in the New Jersey Medicaid program | 6110 | (+65) 100 | 17.0/16.5 | 90.3/84.3 | 1 January 1991 to 31 December 1994 (all 3y) | any use 3 years before index date | F,L,P,S |  |  |
| Woo | 2004 | USA | Case-control | The Genetic and Environmental Risk Factors of Hemorrhagic Stroke study, GERFHS | Participants of the GERFHS | 554 | 65.0 | 50.0 | n.r. | 1 December 1997 to 30 June 2000 (n.r.) | use before index date | n.r. |  |  |
| Yang CC | 2002 | UK | Nested Case-control | General Practice Research Database, GPRD | Primary care patients using GPRD, aged 40-79 years | 504 | (+60) 54.8/54.6 | 50.0 | n.r. | January 1991 to 31 December 1999 (393176person-y) | 1 prescription during the study period | n.r |  |  |
| Yang CC (a) | 2003 | UK | Nested Case-control | General Practice Research Database, GPRD | Primary care patients using GPRD, aged 40-79 years | 2288 | 55.4(9.0) | 53.0 | n.r. | January 1991 to 31 December 1999 (n.r.) | 1 prescription 60 days before index date | A,C,F,P,S |  |  |
| Yang CC (b) | 2003 | UK | Nested Case-control | General Practice Research Database, GPRD | Primary care patients using GPRD, aged 40-79 years | 525 | 55.8(8.5) | 38.1 | n.r. | January 1991 to 31 December 1999 (n.r.) | 1 prescription 60 days before index date | A,C,F,P,S |  |  |
| Yang CC | 2007 | Taiwan | Case-control | Taipei Veterans General Hospital | Patients of Taipei Veterans General Hospital | 719 | 66.2/66.5 | 70.5/72.2 | n.r. | 1 January 1990 to 31 December 2002 (n.r.) | 1 prescription 45 days before index date | n.r |  |  |
| Zamrini | 2004 | USA | Nested Case-control | Veterans Affairs Health Care System, VAHCS | Male patients using VAHCS, 50 years or older | 3397 | 72.9/73.0 | 100 | 55.0/42.3 | 1 January 1997 to 31 December 2001 (n.r.) | 1 prescription 180 days before index date | A,C,F,L,P,S |  |  |
| Zandii | 2005 | USA | Prospective Cohort | The Cache County Study, CCS | Participants of the CCS, 65 years or older | 3308 | 73.0/75.7 | 47.3/42.6 | n.r. | 1995 to 2000 (all 3y) | use at baseline interview | A,C,F,L,P,S |  |  |
| *Age - Mean(SD) for total or per group; or (+60 years) percentage with advanced age ^†^Statin type – A=atorvastatin,C=cerivastatin,F=fluvastatin,P=pravastatin, Pit=pitavastatin, S=simvastatin,R=rosuvastatin | | | | | | | | | | | | |  |  |
| n.r. = not reported: y = year; wk = week; UK = United Kingdom; USA = United States of America; (a) or (b) different studies in the same paper; (1) and (2) different papers of the same author/year | | | | | | | | | | | | | |  |

**Text S1. Search strategy overview.**

| **Database searched** | **Date searched** | **Number of results** |
| --- | --- | --- |
| MEDLINE (OVID) 1946 to January Week 3 2012 | 13/2/12 | 3854 |
| EMBASE & EMBASE Classic (OVID ) 1947 to 2012 Week 06 |  | 8156 |
| **Total** | | **12010** |
| **After de-duplication** | | **10336** |

**Searched run from 1998 onwards**

BMJ Cohort studies filter: <http://clinicalevidence.bmj.com/ceweb/about/search_filters.jsp>

**MEDLINE OVID**

1 exp Hydroxymethylglutaryl-CoA Reductase Inhibitors/ (22021)

2 hydroxymethylglutaryl*.tw. (905)

3 HMG-CoA*.tw. (6335)

4 (statin or statins).tw. (16666)

5 atorvastatin.tw. (3929)

6 cerivastatin.tw. (556)

7 fluvastatin.tw. (1285)

8 lovastatin.tw. (2786)

9 pravastatin.tw. (2889)

10 simvastatin.tw. (5020)

11 lipitor.tw. (115)

12 baycol.tw. (12)

13 lescol.tw. (62)

14 mevacor.tw. (42)

15 altocor.tw. (0)

16 pravachol.tw. (22)

17 lipostat.tw. (24)

18 zocor.tw. (91)

19 mevinolin.tw. (354)

20 compactin.tw. (281)

21 fluindostatin.tw. (4)

22 rosuvastatin.tw. (1165)

23 dalvastatin.tw. (2)

24 cranoc.tw. (0)

25 canef.tw. (0)

26 locol.tw. (0)

27 lochol.tw. (1)

28 leucol.tw. (0)

29 lescol.tw. (62)

30 monacolin.tw. (85)

31 medostatin.tw. (0)

32 mevinacor.tw. (2)

33 livalo.tw. (7)

34 pitava.tw. (2)

35 pitavastatin.tw. (349)

36 pravasin.tw. (0)

37 mevalotin.tw. (8)

38 gerosim.tw. (0)

39 lipex.tw. (5)

40 zenas.tw. (0)

41 crestor.tw. (41)

42 meglutol.tw. (2)

43 or/1-42 (32332)

44 exp Hyperlipidemias/ (51530)

45 exp Cholesterol/ (123867)

46 exp Cardiovascular Diseases/ (1629480)

47 cardio*.tw. (403914)

48 cardia*.tw. (369712)

49 heart*.tw. (532877)

50 coronary*.tw. (254561)

51 angina*.tw. (41290)

52 hyperlipid*.tw. (18280)

53 hypercholesterol*.tw. (22969)

54 cholesterol*.tw. (151911)

55 hypercholester?emia*.tw. (563)

56 hyperlip?emia*.tw. (2074)

57 triglycerid*.tw. (64114)

58 hypertriglycerid?emia*.tw. (7916)

59 hyperlipoprotein?emia*.tw. (4058)

60 LDL.tw. (45266)

61 HDL.tw. (37881)

62 or/44-61 (2302390)

63 43 and 62 (25133)

64 exp cohort studies/ (1136146)

65 cohort$.tw. (184279)

66 controlled clinical trial.pt. (83278)

67 epidemiologic methods/ (27816)

68 limit 67 to yr=1971-1988 (9374)

69 or/64-66,68 (1281187)

70 63 and 69 (4179)

71 (1998* or 1999* or 20*).dc. (8267859)

72 70 and 71 (3854)

**EMBASE OVID**

1 exp hydroxymethylglutaryl coenzyme A reductase inhibitor/ (74067)

2 hydroxymethylglutaryl*.tw. (1015)

3 HMG-CoA*.tw. (8037)

4 (statin or statins).tw. (25725)

5 atorvastatin.tw. (6181)

6 cerivastatin.tw. (718)

7 fluvastatin.tw. (1809)

8 lovastatin.tw. (3631)

9 pravastatin.tw. (3948)

10 simvastatin.tw. (7424)

11 lipitor.tw. (1488)

12 baycol.tw. (264)

13 lescol.tw. (615)

14 mevacor.tw. (742)

15 altocor.tw. (32)

16 pravachol.tw. (575)

17 lipostat.tw. (99)

18 zocor.tw. (1685)

19 mevinolin.tw. (459)

20 compactin.tw. (411)

21 fluindostatin.tw. (4)

22 rosuvastatin.tw. (2078)

23 dalvastatin.tw. (3)

24 cranoc.tw. (34)

25 canef.tw. (24)

26 locol.tw. (46)

27 lochol.tw. (5)

28 leucol.tw. (0)

29 lescol.tw. (615)

30 monacolin.tw. (122)

31 medostatin.tw. (4)

32 mevinacor.tw. (100)

33 livalo.tw. (68)

34 pitava.tw. (6)

35 pitavastatin.tw. (614)

36 pravasin.tw. (101)

37 mevalotin.tw. (100)

38 gerosim.tw. (0)

39 lipex.tw. (52)

40 zenas.tw. (14)

41 crestor.tw. (614)

42 meglutol.tw. (3)

43 or/1-42 (81418)

44 exp cardiovascular disease/ (2778538)

45 cardio*.tw. (585544)

46 cardia*.tw. (531488)

47 heart*.tw. (763669)

48 coronary*.tw. (351750)

49 angina*.tw. (59364)

50 hyperlipidemia/ (44992)

51 exp cholesterol/ (198011)

52 exp lipid blood level/ (117947)

53 hyperlipid*.tw. (25980)

54 hypercholesterol*.tw. (32076)

55 cholesterol*.tw. (209139)

56 hypercholester?emia*.tw. (984)

57 hyperlip?emia*.tw. (3923)

58 triglycerid*.tw. (89160)

59 hypertriglycerid?emia*.tw. (10689)

60 hyperlipoprotein?emia*.tw. (5837)

61 LDL.tw. (61161)

62 HDL.tw. (52642)

63 or/44-62 (3553033)

64 43 and 63 (65249)

65 exp cohort analysis/ (109470)

66 exp longitudinal study/ (49047)

67 exp prospective study/ (183476)

68 exp follow up/ (602568)

69 cohort$.tw. (253329)

70 or/65-69 (988002)

71 64 and 70 (8769)

72 (animal/ or nonhuman/) not human/ (4350497)

73 (1998* or 1999* or 20*).em. (11090714)

74 71 not 72 (8760)

75 limit 74 to embase (8377)

76 73 and 75 (8256)

**Text S2. Study references.**

**86 References included (4 articles reported two different studies, accounting for a total of 90 studies)**

1. [Anderson JL](http://www.ncbi.nlm.nih.gov/pubmed?term=Anderson%20JL%5BAuthor%5D&cauthor=true&cauthor_uid=15842981), [Muhlestein JB](http://www.ncbi.nlm.nih.gov/pubmed?term=Muhlestein%20JB%5BAuthor%5D&cauthor=true&cauthor_uid=15842981), [Bair TL](http://www.ncbi.nlm.nih.gov/pubmed?term=Bair%20TL%5BAuthor%5D&cauthor=true&cauthor_uid=15842981), [Morris S](http://www.ncbi.nlm.nih.gov/pubmed?term=Morris%20S%5BAuthor%5D&cauthor=true&cauthor_uid=15842981), [Weaver AN](http://www.ncbi.nlm.nih.gov/pubmed?term=Weaver%20AN%5BAuthor%5D&cauthor=true&cauthor_uid=15842981), [Lappé DL](http://www.ncbi.nlm.nih.gov/pubmed?term=Lapp%C3%A9%20DL%5BAuthor%5D&cauthor=true&cauthor_uid=15842981), [Renlund DG](http://www.ncbi.nlm.nih.gov/pubmed?term=Renlund%20DG%5BAuthor%5D&cauthor=true&cauthor_uid=15842981), [Pearson RR](http://www.ncbi.nlm.nih.gov/pubmed?term=Pearson%20RR%5BAuthor%5D&cauthor=true&cauthor_uid=15842981), [Jensen KR](http://www.ncbi.nlm.nih.gov/pubmed?term=Jensen%20KR%5BAuthor%5D&cauthor=true&cauthor_uid=15842981), [Horne BD](http://www.ncbi.nlm.nih.gov/pubmed?term=Horne%20BD%5BAuthor%5D&cauthor=true&cauthor_uid=15842981). Do statins increase the risk of idiopathic polyneuropathy? [Am J Cardiol.](http://www.ncbi.nlm.nih.gov/pubmed/15842981) 2005 May 1;95(9):1097-9.
2. Ashfield TA, Syddall HE, Martin HJ, Dennison EM, Cooper C, Aihie Sayer A. Grip strength and cardiovascular drug use in older people: findings from the Hertfordshire Cohort Study. Age Ageing. 2010;39(2):185-91.
3. Atthobari J, Brantsma AH, Gansevoort RT, Visser ST, Asselbergs FW, van Gilst WH, et al. The effect of statins on urinary albumin excretion and glomerular filtration rate: results from both a randomized clinical trial and an observational cohort study. Nephrol Dial Transplant. 2006;21(11):3106-14.
4. Becker C, Jick SS, Meier CR, Becker C, Jick SS, Meier CR. Use of statins and the risk of Parkinson's disease: a retrospective case-control study in the UK. Drug Safety. 2008;31(5):399-407.
5. Bernick C, Katz R, Smith NL, Rapp S, Bhadelia R, Carlson M, et al. Statins and cognitive function in the elderly: the Cardiovascular Health Study. Neurology. 2005;65(9):1388-94.
6. Chan KA, Andrade SE, Boles M, Buist DS, Chase GA, Donahue JG, Goodman MJ, Gurwitz JH, LaCroix AZ, Platt R. Inhibitors of hydroxymethylglutaryl-coenzyme A reductase and risk of fracture among older women. Lancet. 2000 Jun 24;355(9222):2185-8.
7. Clockaerts S, Stricker B, Bastiaansen-Jenniskens YM, Van Glabbeek F, Van Meurs JB, Verhaar JA, et al. Statin use is associated with reduced incidence and progression of knee osteoarthritis. Osteoarthritis and Cartilage. 2010;18:S38.
8. Corrao G, Zambon A, Bertù L, Botteri E, Leoni O, Contiero P. Lipid lowering drugs prescription and the risk of peripheral neuropathy: an exploratory case-control study using automated databases. J Epidemiol Community Health. 2004 Dec;58(12):1047-51.
9. Cramer C, Haan MN, Galea S, Langa KM, Kalbfleisch JD. Use of statins and incidence of dementia and cognitive impairment without dementia in a cohort study. Neurology. 2008;71(5):344-50.
10. Culver AL, Ockene IS, et al. [Statin use and risk of diabetes mellitus in postmenopausal women in the Women's Health Initiative.](http://www.ncbi.nlm.nih.gov/pubmed/22231607) Arch Intern Med. 2012 Jan 23;172(2):144-52.
11. de Jong HJ, Klungel OH, van Dijk L, et al. [Use of statins is associated with an increased risk of rheumatoid arthritis.](http://www.ncbi.nlm.nih.gov/pubmed/21979000) Ann Rheum Dis. 2012 May;71(5):648-54.
12. de Vries F, de Vries C, Cooper C, Leufkens B, van Staa TP. Reanalysis of two studies with contrasting results on the association between statin use and fracture risk: the General Practice Research Database. Int J Epidemiol. 2006 Oct;35(5):1301-8.
13. Doggen CJM, Lemaitre RN, Smith NL, Heckbert SR, Psaty BM. HMG CoA reductase inhibitors and the risk of venous thrombosis among post-menopausal women. J Thromb Haemost 2003;2:700–701.
14. Erichsen R, Frøslev T, et al. [Long-term statin use and the risk of gallstone disease: A population-based case-control study.](http://www.ncbi.nlm.nih.gov/pubmed/21084557) Am J Epidemiol. 2011 Jan 15;173(2):162-70.
15. Fleming DM, Verlander NQ, Elliot AJ, Zhao H, Gelb D, Jehring D, et al. An assessment of the effect of statin use on the incidence of acute respiratory infections in England during winters 1998-1999 to 2005-2006. Epidemiol Infect. 2010;138(9):1281-8.
16. Fujii T, Nakabayashi T, Hashimoto S, Kuwano H. Statin use and risk of gastroduodenal ulcer and reflux esophagitis. Hepatogastroenterology. 2009;56(91-92):641-4.
17. Gaist D, Garcia Rodriguez LA, Huerta C, Hallas J, Sindrup SH. Are users of lipid-lowering drugs at increased risk of peripheral neuropathy? Eur J Clin Pharmacol. 2001;56(12):931-3.
18. Gaist D, Jeppesen U, Andersen M, García Rodríguez LA, Hallas J, Sindrup SH. Statins and risk of polyneuropathy: a case-control study. Neurology. 2002 May 14;58(9):1333-7.
19. Gaist D, Rodriguez LA, Huerta C, Hallas J, Sindrup SH. Lipid-lowering drugs and risk of myopathy: a population-based follow-up study. Epidemiology. 2001;12(5):565-9.
20. Gao X, Simon K, Schwarzschild MA, Ascherio A. A prospective study of statin use and risk of Parkinson disease. Parkinsonism and Related Disorders. 2012;18:S40.
21. Green RC, McNagny SE, Jayakumar P, Cupples LA, Benke K, Farrer LA; MIRAGE Study Group. Statin use and the risk of Alzheimer's disease: the MIRAGE study. Alzheimers Dement. 2006 Apr;2(2):96-103.
22. Gulmez SE, Lassen AT, Aalykke C, Dall M, Andries A, Andersen BS, Hansen JM, Andersen M, Hallas J. [Do statins protect against upper gastrointestinal bleeding?](http://www.ncbi.nlm.nih.gov/pubmed/19371320) Br J Clin Pharmacol. 2009 Apr;67(4):460-5.
23. Haag MDM, Hofman A, Koudstaal PJ, Stricker BHC, Breteler MMB. Statins are associated with a reduced risk of Alzheimer disease regardless of lipophilicity. The Rotterdam Study. J Neurol Neurosurg Psychiatry. 2009;80(1):13-7.
24. Hippisley-Cox J, Coupland C. Unintended effects of statins in men and women in England and Wales: population based cohort study using the QResearch database. Bmj. 2010;340:c2197.
25. Huang X, Chen H, Miller WC, Mailman RB, Woodard JL, Chen PC, et al. Lower low-density lipoprotein cholesterol levels are associated with Parkinson's disease. Need to read full paper. Mov Disord. 2007;22(3):377-81.
26. Huerta C, Johansson S, Wallander MA, Garcıa Rodrıguez LA. Risk factors and short-term mortality of venous thromboembolism diagnosed in the primary care setting in the United Kingdom. Arch Intern Med 2007;167:935–943.
27. Jick H, Zornberg GL, Jick SS, Seshadri S, Drachman DA. Statins and the risk of dementia. Lancet Nov 2000 11;356:1627–31.
28. Jick SS, Bradbury BD. [Statins and newly diagnosed diabetes.](http://www.ncbi.nlm.nih.gov/pubmed/15327590) Br J Clin Pharmacol. 2004 Sep;58(3):303-9.
29. Jick SS, Choi H, Li L, McInnes IB, Sattar N. Hyperlipidaemia, statin use and the risk of developing rheumatoid arthritis. Ann Rheum Dis. 2009 Apr;68(4):546-51.
30. Klein BE, Klein R, Lee KE, et al. Statin use and incident nuclear cataract. JAMA 2006; 295: 2752-8.
31. Klein R, Klein BEK, Tomany SC, Danforth LG, Cruickshanks KJ, Rumelt S. Relation of statin use to the 5-year incidence and progression of age-related maculopathy. Evidence-Based Eye Care. 2004;5(2):70-1.
32. Klein R, Knudtson MD, Klein BE. [Statin use and the five-year incidence and progression of age-related macular degeneration.](http://www.ncbi.nlm.nih.gov/pubmed/17475196) Am J Ophthalmol. 2007 Jul;144(1):1-6.
33. Kwong JC, Li P, Redelmeier DA. [Influenza morbidity and mortality in elderly patients receiving statins: a cohort study.](http://www.ncbi.nlm.nih.gov/pubmed/19956645) PLoS One. 2009 Nov 30;4(11):e8087.
34. LaCroix AZ, Cauley JA, Pettinger M, et al. Statin use, clinical fracture, and bone density in postmenopausal women: results from the Women’s Health Initiative Observational Study. Ann Intern Med 2003; 139 (2): 97-104.
35. LaCroix AZ, Gray SL, Aragaki A, Cochrane BB, Newman AB, Kooperberg CL, et al. Statin use and incident frailty in women aged 65 years or older: prospective findings from the Women's Health Initiative Observational Study. J Gerontol A Biol Sci Med Sci. 2008;63(4):369-75.
36. Lacut K, Le Gal G, Abalain JH, et al. Differential associations between lipid-lowering drugs, statins and fibrates, and venous thromboembolism: role of drug induced homocysteinemia? Thromb Res 2008;122:314–319
37. Lemaitre RN, Psaty BM, Heckbert SR, Kronmal RA, Newman AB, Burke GL. Therapy with hydroxymethylglutaryl coenzyme a reductase inhibitors (statins) and associated risk of incident cardiovascular events in older adults: evidence from the Cardiovascular Health Study. Arch Intern Med. 2002;162(12):1395-400.
38. Li G, Higdon R, Kukull WA, Peskind E, Van Valen Moore K, Tsuang D, et al. Statin therapy and risk of dementia in the elderly: A community-based prospective cohort study. Neurology. 2004;63(9):1624-8.
39. Li G, Shofer JB, Rhew IC, Kukull WA, Peskind ER, McCormick W, et al. Age-varying association between statin use and incident Alzheimer's disease. J Am Geriatr Soc. 2010;58(7):1311-7.
40. Luijendijk HJ, Stricker BH, Hofman A, Witteman JCM, Tiemeier H. Cerebrovascular risk factors and incident depression in community-dwelling elderly. Acta Psychiatr Scand. 2008;118(2):139-48.
41. Marcus MW, Muskens RPHM, Ramdas WD, Wolfs RCW, de Jong PTVM, Vingerling JR, et al. Cholesterol-lowering drugs and incident open-angle glaucoma: A population-based cohort study. PLoS ONE [Electronic Resource]. 2012;7(1).
42. McClure DL, Valuck RJ, Glanz M, Murphy JR, Hokanson JE, McClure DL, et al. Statin and statin-fibrate use was significantly associated with increased myositis risk in a managed care population. Journal of Clinical Epidemiology. 2007;60(8):812-8.
43. McGwin G Jr, Owsley C, Curcio CA, et al.The association between statin use and age related maculopathy. Br J Ophthalmol 2003 87:1121–5.
44. McGwin G, Jr., McNeal S, Owsley C, Girkin C, Epstein D, et al. Statins and other cholesterol-lowering medications and the presence of glaucoma. Arch Ophthalmol 2004 122: 822–826.
45. McGwin G, Modjarrad K, Andrew Hal T, Xie A, Owsley C. 3-Hydroxy-3-Methylglutaryl Coenzyme A Reductase Inhibitors and the Presence of Age-Related Macular Degeneration in the Cardiovascular Health Study. Arch Ophthalmol. 2006;124:33-37.
46. Meier CR, Schlienger RG, Kraenzlin ME, Schlegel B, Jick H. HMG-CoA reductase inhibitors and the risk of fractures. *JAMA.* 2000;283:3205-3210.
47. Myles PR, Hubbard RB, McKeever TM, Pogson Z, Smith CJ, Gibson JE. [Risk of community-acquired pneumonia and the use of statins, ace inhibitors and gastric acid suppressants: a population-based case-control study.](http://www.ncbi.nlm.nih.gov/pubmed/19235776) Pharmacoepidemiol Drug Saf. 2009 Apr;18(4):269-75.
48. Nash SD, Cruickshanks KJ, Klein R, Klein BEK, Nieto FJ, Huang GH, et al. The prevalence of hearing impairment and associated risk factors: the Beaver Dam Offspring Study. Arch Otolaryngol Head Neck Surg. 2011;137(5):432-9.
49. Nichols GA, Koro CE. Does statin therapy initiation increase the risk for myopathy? An observational study of 32,225 diabetic and nondiabetic patients. Clin Ther. 2007;29(8):1761-70.
50. Owen CG, Carey IM, Shah S, de Wilde S, Wormald R, Whincup PH, Cook DG.Hypotensive medication, statins, and the risk of glaucoma.Invest Ophthalmol Vis Sci. 2010 Jul;51(7):3524-30.
51. Ramcharan AS, van Stralen KJ, Mantel-Teeuwisse AK, et al. HMG-CoA reductase inhibitors are associated with a reduced risk of venous thrombosis. J Thromb Haemost 2009;7:514–520.
52. Ray WA, Daugherty JR, Griffin MR. Lipid-lowering agents and the risk of hip fracture in a Medicaid population. Inj Prev. 2002;8(4):276-9.
53. Rea TD, Breitner JC, Psaty BM, Fitzpatrick AL, Lopez OL, Newman AB, et al. Statin use and the risk of incident dementia: the Cardiovascular Health Study. Archives of Neurology. 2005;62(7):1047-51.
54. Rejnmark L, Olsen ML, Johnsen SP, et al. Hip fracture risk in statin users-a population-based Danish case-control study. Osteoporos Int 2004;15:452–8
55. Risselada R, Straatman H, van Kooten F, Dippel DWJ, van der Lugt A, Niessen WJ, et al. Withdrawal of statins and risk of subarachnoid hemorrhage. Stroke. 2009;40(8):2887-92.
56. Ritz B, Manthripragada AD, Qian L, Schernhammer E, Wermuth L, Olsen J, Friis S. [Statin use and Parkinson's disease in Denmark.](http://www.ncbi.nlm.nih.gov/pubmed/20629142) Mov Disord. 2010 Jul 15;25(9):1210-6.
57. Rockwood K, Howlett S, Fisk J, Darvesh S, Tuokko H, Hogan DB, et al. Lipid-lowering agents and the risk of cognitive impairment that does not meet criteria for dementia, in relation to apolipoprotein E status. Neuroepidemiology. 2007;29(3-4):201-7.
58. Rockwood K, Kirkland S, Hogan DB, MacKnight C, Merry H, Verreault R, et al. Use of lipid-lowering agents, indication bias, and the risk of dementia in community-dwelling elderly people. Archives of Neurology. 2002;59(2):223-7.
59. [Rodríguez GL](http://www.ncbi.nlm.nih.gov/pubmed?term=Garc%C3%ADa%20Rodr%C3%ADguez%20LA%5BAuthor%5D&cauthor=true&cauthor_uid=19863366), [Wallander MA](http://www.ncbi.nlm.nih.gov/pubmed?term=Wallander%20MA%5BAuthor%5D&cauthor=true&cauthor_uid=19863366), [Tolosa LB](http://www.ncbi.nlm.nih.gov/pubmed?term=Tolosa%20LB%5BAuthor%5D&cauthor=true&cauthor_uid=19863366), [Johansson S](http://www.ncbi.nlm.nih.gov/pubmed?term=Johansson%20S%5BAuthor%5D&cauthor=true&cauthor_uid=19863366). Chronic obstructive pulmonary disease in UK primary care: incidence and risk factors. [COPD.](http://www.ncbi.nlm.nih.gov/pubmed/19863366) 2009 Oct;6(5):369-79.
60. Schlienger RG, Fedson DS, Jick SS, Jick H, Meier CR. Statins and the risk of pneumonia: a population-based, nested case-control study. Pharmacotherapy. 2007;27(3):325-32.
61. Schlienger RG, Haefeli WE, Hershel J, et al. [Risk of cataract in patients treated with statins.](http://www.ncbi.nlm.nih.gov/pubmed/11525705) Arch Intern Med. 2001 Sep 10;161(16):2021-6
62. Schoofs MWCJ, Sturkenboom MCJM, van der Klift M, Hofman A, Pols HAP, Stricker BHC. HMG-CoA reductase inhibitors and the risk of vertebral fracture. J Bone Miner Res. 2004;19(9):1525-30.
63. Scranton RE , Young M , Lawler L , Solomon DH , Gagnon DR , Gaziano JM . Statin use is associated with fewer fractures: study from a US veterans population . Arch Intern Med *.* 2005 ; 165 : 2007 – 2012
64. Smeeth L, Cook C, Chakravarthy U, Hubbard R, Fletcher AE. A case control study of age related macular degeneration and use of statins. Br J Ophthalmol. 2005 Sep;89(9):1171-5.
65. Smeeth L, Douglas I, Hall AJ, Hubbard R, Evans S. Effect of statins on a wide range of health outcomes: a cohort study validated by comparison with randomized trials. Br J Clin Pharmacol. 2009;67(1):99-109.
66. Smeeth L, Hubbard R, Fletcher AE. Cataract and the use of statins: a case-control study. QJM. 2003 May;96(5):337-43. PubMed PMID: 12702782.
67. Solomon A, Kareholt I, Ngandu T, Wolozin B, MacDonald SWS, Winblad B, et al. Serum total cholesterol, statins and cognition in non-demented elderly. Neurobiology of Aging. 2009;30(6):1006-9.
68. Sørensen HT, Horvath-Puho E, Søgaard KK, Christensen S, Johnson SP, Thomsen RW, Prandoni P, Baron JA. Arterial cardiovascular events, statins, low dose aspirin and subsequent risk of venous thromboembolism: a populationbased case–control study. J Thromb Haemost 2009;7:521–528.
69. Sorensen HT, Riis AH, Lash TL, Pedersen L. Statin use and risk of amyotrophic lateral sclerosis and other motor neuron disorders. Circ Cardiovasc Qual Outcomes. 2010;3(4):413-7.
70. St Sauver JL, Jacobsen SJ, Jacobson DJ, McGree ME, Girman CJ, Nehra A, et al. Statin use and decreased risk of benign prostatic enlargement and lower urinary tract symptoms. BJU Int. 2011;107(3):443-50.
71. Sukhija R, Bursac Z, Kakar P, Fink L, Fort C, Satwani S, et al. Effect of Statins on the Development of Renal Dysfunction. Am J Cardiol. 2008;101(7):975-9.
72. Szwast SJ, Hendrie HC, Lane KA, Gao S, Taylor SE, Unverzagt F, et al. Association of statin use with cognitive decline in elderly African Americans. Neurology. 2007;69(19):1873-80.
73. Tan JS, Mitchell P, Rochtchina E, Wang JJ (2007) Statin use and the long-term risk of incident cataract: The blue mountains eye study. Am J Ophthalmol 143: 687–689.
74. Tan JS, Mitchell P, Rochtchina E, Wang JJ. Statins and the long-term risk of incident age-related macular degeneration: the Blue Mountains Eye Study. Am J Ophthalmol. 2007 Apr;143(4):685-7.
75. Tsai C-J, Leitzmann MF, Willett WC, Giovannucci EL. Statin use and the risk of cholecystectomy in women. Gastroenterology. 2009;136(5):1593-600.
76. van Leeuwen R, Vingerling JR, Hofman A, et al. 2003. Cholesterol lowering drugs and risk of age related maculopathy: prospective cohort study with cumulative exposure measurement. BMJ, 326:255–6.
77. van LeeuwenR, Tomany SC,Wang JJ, et al. Is medication use associated with the incidence of early age-related maculopathy? Pooled findings from 3 continents. Ophthalmology 2004; 111 (6): 1169-75
78. van Staa T, Wegman S, de Vries F, Leufkens B, Cooper C. Use of statins and risk of fractures. *JAMA.* 2001;285:1850-1855.
79. Wahner AD, Bronstein JM, Bordelon YM, Ritz B. Statin use and the risk of Parkinson disease. Neurology. 2008 Apr 15;70(16 Pt 2):1418-22.
80. Wang PS, Solomon DH, Mogun H, Avorn J. HMG-CoA reductase inhibitors and the risk of hip fractures in elderly patients. JAMA*.* 2000;283:3211-3216
81. Woo D, Kissela BM, Khoury JC, Sauerbeck LR, Haverbusch MA, Szaflarski JP, Gebel JM, Pancioli AM, Jauch EC, Schneider A, Kleindorfer D, Broderick JP. Hypercholesterolemia, HMG-CoA reductase inhibitors, and risk of intracerebral hemorrhage: a case-control study. Stroke. 2004 Jun;35(6):1360-4.
82. Yang CC, Jick SS, Jick H. Lipid-lowering drugs and the risk of depression and suicidal behavior. Arch Intern Med. 2003 Sep 8;163(16):1926-32.
83. Yang C-C, Jick SS, Jick H. Statins and the risk of idiopathic venous thromboembolism. Br J Clin Pharmacol. 2002;53(1):101-5.
84. Yang CC, Kao CC. Cardiovascular diseases and the risk of venous thromboembolism: a hospital-based case-control study. J Chin Med Assoc. 2007 Mar;70(3):103-9.
85. Zamrini E, McGwin G, Roseman JM. Association between statin use and Alzheimer's disease. Neuroepidemiology. 2004 Jan-Apr;23(1-2):94-8.
86. Zandi PP, Sparks DL, Khachaturian AS, et al; Cache County Study investigators. Do statins reduce risk of incident dementia and Alzheimer disease? The Cache County Study. Arch Gen Psychiatry. 2005 Feb;62(2):217-24.

**5 References awaiting classification**

1. Agarwal SJ, Johnson ML. The association of co-medications and co-morbidities and risk of dementia, in patients with chronic heart failure. Value in Health. 2009;12 (3):A141.
2. Hake AM, Gao S, Lane K, Unverzagt F, Smith-Gamble V, Murrell J, et al. Statin use and incident dementia and alzheimer's disease in elderlyafricanamericans. Alzheimer's and Dementia. 2011;1):S511-S2
3. Li L, Thompson C, Tucker T: No association between lipid-lowering statin use and risk of colon cancer. Presented at 34th Annual Meeting of the North American Primary Care Research Group (NAPCRG), October 15-18, 2006, abstr CC7.
4. Misra A, Hansen LG, Chang S. Periodontal disease, statin use, and cardiovascular events. Consider subgroup of patients? Read full paper... Value in Health. 2009;12 (7):A312.
5. Naik HB, Han J, Li T, Qureshi AA. Statin use is not protective for incident psoriasis. Journal of Investigative Dermatology. 2011;131:S40.

**63 References excluded**

1. Ahern TP, Pedersen L, Tarp M, Cronin-Fenton DP, Garne JP, Silliman RA, et al. Statin prescriptions and breast cancer recurrence risk: a Danish nationwide prospective cohort study. J Natl Cancer Inst. 2011;103(19):1461-8.
2. Akduman B, Tandberg DJ, O'Donnell C, Hughes A, Moyad MA, Crawford ED. Association of statin use to Serum prostate specific antigen levels in a screening population. Journal of Urology. 2010;1):e715-e6.
3. Akduman B, Tandberg DJ, O'Donnell CI, Hughes A, Moyad MA, Crawford ED. Effect of Statins on Serum Prostate-specific Antigen Levels. Urology. 2010;76(5):1048-51.
4. Almog Y, Novack V, Eisinger M, Porath A, Novack L, Gilutz H. The effect of statin therapy on infection-related mortality in patients with atherosclerotic diseases. Crit Care Med. 2007;35(2):372-8.
5. Amital H, Chodick G, Shalem Y, Shalev V. HMG-CoA reductase inhibitors (statins) provides primary prevention for rheumatoid arthritis. Arthritis and Rheumatism. 2009;60:1174.
6. Arvanitakis Z, Schneider JA, Wilson RS, Bienias JL, Kelly JF, Evans DA, et al. Statins, incident Alzheimer disease, change in cognitive function, and neuropathology. Neurology. 2008;70(19 Pt 2):1795-802.
7. Avins AL, Manos MM, Ackerson L, Zhao W, Murphy R, Levin TR, et al. Hepatic effects of lovastatin exposure in patients with liver disease: a retrospective cohort study. Drug Safety. 2008;31(4):325-34.
8. Berard E, Bongard V, Amouyel P, Arveiler D, Dallongeville J, Wagner A, et al. Ten-year risk of cancer mortality according to lipid levels and use of lipid-lowering drugs in the French general population. Eur Heart J. 2009;30:148.
9. Berard E, Bongard V, Amouyel P, Arveiler D, Dallongeville J, Wagner A, et al. Ten-year risk of cancer mortality according to lipid levels and use of lipid-lowering drugs in the French general population. Archives of Cardiovascular Diseases Supplements. 2010;2 (1):91-2.
10. Bhutta HY, Clark A, Holt S, Lewis MPN, Hart AR. Oesophageal cancer - An aetiological investigation into the potential protective effect of statins in the uk general practice research database (GPRD). Gut. 2011;60:A36-A7.
11. Biere-Rafi S, Gerdes VEA, Hutten BA, Squizzato A, Ageno W, Souverein PC, et al. Statin treatment for primary and secondary prevention of pulmonary embolism: A population based case-control study. Pathophysiology of Haemostasis and Thrombosis. 2010;37:A1.
12. Bushnell CD, Newby LK, Goldstein LB, Lin F, Yaffe K, Simon JA. Statin use and stroke outcomes in the Heart and Estrogen-Progestin Replacement Study (HERS). Neurology. 2004;62(6):968-70.
13. Carlsson CM, Nondahl DM, Klein BEK, McBride PE, Sager MA, Schubert CR, et al. Increased atherogenic lipoproteins are associated with cognitive impairment: effects of statins and subclinical atherosclerosis. Alzheimer Dis Assoc Disord. 2009;23(1):11-7.
14. Chodick G, Amital H, Shalem Y, Kokia E, Heymann AD, Porath A, et al. Persistence with statins and onset of rheumatoid arthritis: A population-based cohort study. Possible wrong comparators! PLoS Medicine. 2010;7(9).
15. Chodick G, Heymann A, Flash S, Kokia E, Shalev V. Persistence with statins and incident cataract: a population-based historical cohort study. Ann Epidemiol 2010 20: 136–142.
16. Chodick G, Shalev V, Goldstein I, Porath A, Simah V. Persistence with statins and primary prevention of cardiovascular events: A population-based cohort study. Value in Health. 2011;14 (3):A35-A6.
17. Dufouil C, Richard F, Fievet N, Dartigues JF, Ritchie K, Tzourio C, et al. APOE genotype, cholesterol level, lipid-lowering treatment, and dementia: the Three-City Study. Neurology. 2005;64(9):1531-8.
18. Edwards CJ, Hart DJ, Spector TD 2000 Oral statins and increased bone-mineral density in postmenopausal women. Lancet **355:** 2218–2219.
19. Elizondo C, Posadas L, Berecoechea C, Giunta D, Fuentes N, Pazo V, et al. Incidence of thomboembolic venous disease and statins use. Internal Medicine Journal. 2010;40:15.
20. Farwell WR, D'Avolio LW, Scranton RE, Lawler EV, Gaziano JM. Statins and prostate cancer diagnosis and grade in a veterans population. J Natl Cancer Inst. 2011;103(11):885-92.
21. Feng L, Yap KB, Kua EH, Ng TP. Statin use and depressive symptoms in a prospective study of community-living older persons. Pharmacoepidemiol Drug Saf. 2010;19(9):942-8.
22. Gopinath B, Flood VM, Teber E, McMahon CM, Mitchell P. Dietary intake of cholesterol is positively associated and use of cholesterol-lowering medication is negatively associated with prevalent age-related hearing loss. J Nutr. 2011;141(7):1355-61.
23. GrahamDJ, Staffa JA, Shatin D, Andrade SE, Schech SD, LaGrenade L, et al. Incidence of hospitalized rhabdomyolysis in patients treated with lipid-lowering drugs. JAMA 2004;292:2585-90.
24. Hackam DG, Austin PC, Huang A, Juurlink DN, Mamdani MM, Paterson JM, et al. Statins and intracerebral hemorrhage: A retrospective cohort study. Archives of Neurology. 2012;69(1):39-45.
25. Hackam DG, Wu F, Li P, Austin PC, Tobe SW, Mamdani MM, et al. Statins and renovascular disease in the elderly: a population-based cohort study. Eur Heart J. 2011;32(5):598-610.
26. Hajjar I, Schumpert J, Hirth V, Wieland D, Eleazer GP. The impact of the use of statins on the prevalence of dementia and the progression of cognitive impairment. J Gerontol A Biol Sci Med Sci. 2002;57(7):M414-8.
27. Hall SA, Chiu GR, Steers WD, Link CL, McKinlay JB. Do statins improve urologic symptoms? Journal of Urology. 2011;1):e692.
28. Hall SA, Page ST, Travison TG, et al. Do statins affect androgen levels in men? Results from the Boston area community health survey. Cancer Epidemiol Biomarkers Prev. 2007;16:1587-1594
29. Heintjes EM, Penning-van Beest FJA, Johansson S, Stalenhoef AF, Herings RMC. Comparison of incidences of cardiovascular events among new users of different statins: a retrospective observational cohort study. Curr Med Res Opin. 2009;25(11):2621-9.
30. Hodgkinson JA, Taylor CJ, Hobbs FDR. Predictors of incident atrial fibrillation and influence of medications: a retrospective case-control study. Br J Gen Pract. 2011;61(587):e353-61.
31. Ishida W, Kajiwara T, Ishii M, et al. Decrease in mortality rate of chronic obstructive pulmonary disease (COPD) with statin use: a population-based analysis in Japan. Tohoku J Exp Med 2007; 212:265–273
32. Itakura H, Nakaya N, Kusunoki T, Shimizu N, Hirai S, Mochizuki S, et al. Long-term event monitoring study of fluvastatin in Japanese patients with hypercholesterolemia: Efficacy and incidence of cardiac and other events in elderly patients (>= 65 years old). J Cardiol. 2011;57(1):77-88.
33. Jacobs EJ, Newton CC, Thun MJ, Gapstur SM. Long-term use of cholesterol-lowering drugs and cancer incidence in a large United States cohort. Cancer Prevention Research Conference: AACR International Conference on Frontiers in Cancer Prevention Research Philadelphia, PA United States Conference Start. 2010;3(12 SUPPL. 2).
34. Jong GP, Ma T, Tien L. Long-term effect of statins on the risk of new-onset diabetes - A retrospective cohort study. Eur Heart J. 2010;31:530.
35. Khemasuwan D, Chae YK, Gupta S. Dose-related effect of statins in venous thrombosis risk reduction. Hospital based study - exclude? Journal of Vascular Surgery. 2012;55 (2):614-5.
36. Khurana V, Jaganmohan S, Chalasani R, et al: Statins do not reduce colon cancer risk in humans: A case-control study in half million veterans. Am J Gastroenterol 99:242s, 2004; abstr 746.
37. Klein R, Klein BE, Jensen SC, et al. Medication use and the 5-year incidence of early age-related maculopathy: the Beaver Dam Eye Study. Arch Ophthalmol. 2001; 119:1354-1359.
38. Li G, Larson EB. Do statins lower risk of dementia and Alzheimer's disease? 2006. p. 160-5.
39. Masse I, Bordet R, Deplanque D, Al Khedr A, Richard F, Libersa C, Pasquier F: Lipid lowering agents are associated with a slower cognitive decline in Alzheimer’s disease. J Neurol Neurosurg Psychiatry 2005; 76: 1624– 1629
40. McCarty CA, Mukesh BN, Fu CL, et al. 2001. Risk factors for age-related maculopathy. The Visual Impairment Project. Arch Ophthalmol. 2001,119:1455-1462.
41. McGwin G, Jr., Xie A, Owsley C. The use of cholesterol-lowering medications and age-related macular degeneration. Ophthalmology. 2005;112(3):488-94.
42. Mehta JL, Bursac Z, Hauer-Jensen M, Fort C, Fink LM. Comparison of Mortality Rates in Statin Users Versus Nonstatin Users in a United States Veteran Population. Am J Cardiol. 2006;98(7):923-8.
43. Molokhia M, McKeigue P, Curcin V, Majeed A, Molokhia M, McKeigue P, et al. Statin induced myopathy and myalgia: time trend analysis and comparison of risk associated with statin class from 1991-2006. PLoS ONE [Electronic Resource]. 2008;3(6):e2522.
44. Mondul AM, Selvin E, De Marzo AM, Freedland SJ, Platz EA. Statin drugs, serum cholesterol, and prostate-specific antigen in the National Health and Nutrition Examination Survey 2001-2004. Cancer Causes Control. 2010 May;21(5):671-8.
45. Mutez E, Duhamel A, Defebvre L, Bordet R, Destee A, Kreisler A. Lipid-lowering drugs are associated with delayed onset and slower course of Parkinson's disease. Pharmacol Res. 2009;60(1):41-5.
46. Nehra, A., J. L. St. Sauver, Jacobson D, et al. Statin use and development of erectile dysfunction. Journal of Urology 2009; 181(4): 330.
47. Olsen JH, Johansen C, Sørensen HT, McLaughlin JK, Mellemkjaer L, Steffensen FH, Fraumeni JF Jr. Lipid-lowering medication and risk of cancer. J Clin Epidemiol. 1999 Feb;52(2):167-9.
48. Ray JG, Mamdani M, Tsuyuki RT, Anderson DR, Yeo EL, Laupacis A. Use of statins and the subsequent development of deep vein thrombosis. Arch Intern Med. 2001;161(11):1405-10.
49. Rodriguez EG, Dodge HH, Birzescu MA, Stoehr GP, Ganguli M. Use of lipid-lowering drugs in older adults with and without dementia: a community-based epidemiological study. J Am Geriatr Soc. 2002;50(11):1852-6.
50. Rubin DT, Blumentals WA, Sheer RL, et al: Statins and risk of colorectal cancer: Results from a large case-control study. Am J Gastroenterol 2005; 100(394s); abstr 1086.
51. Sakabe K, Fukuda N, Wakayama K, Nada T, Shinohara H, Tamura Y. Lipid-altering changes and pleiotropic effects of atorvastatin in patients with hypercholesterolemia. Am J Cardiol. 2004;94(4):497-500.
52. Setoguchi S, Glynn RJ, Avorn J, Mogun H, Schneeweiss S. Statins and the risk of lung, breast, and colorectal cancer in the elderly. Circulation. 2007;115(1):27-33.
53. Setoguchi S. Statins and cancer in the elderly. Cardiology Review. 2007;24(9):13-6.
54. Shalev V, Chodick G, Silber H, Kokia E, Jan J, Heymann AD. Continuation of statin treatment and all-cause mortality: a population-based cohort study. Arch Intern Med. 2009;169(3):260-8.
55. Shalev V, Sror M, Goldshtein I, Kokia E, Chodick G. Statin use and the risk of age related macular degeneration in a large health organization in Israel. Ophthalmic Epidemiol. 2011;18(2):83-90.
56. Silveira MJ, Kazanis AS, Shevrin MP. Statins in the last six months of life: A recognizable, life-limiting condition does not decrease their use. Journal of Palliative Medicine. 2008;11(5):685-93.
57. Solomon A, Kreholt I, Ngandu T, Winblad B, Nissinen A, Tuomilehto J, et al. Risk factor versus risk marker: Serum total cholesterol, its changes after midlife and late-life cognitive impairment. Journal of the Neurological Sciences. 2009;283 (1-2):258.
58. Solomon A, Soininen H, Laatikainen T, Tuomilehto J, Kivipelto M. Statins and dementia prevention: A population-based study (FINRISK). Alzheimer's and Dementia. 2009;1):292.
59. Starr JM, McGurn B, Whiteman M, Pattie A, Whalley LJ, Deary IJ, et al. Life long changes in cognitive ability are associated with prescribed medications in old age. International Journal of Geriatric Psychiatry. 2004;19(4):327-32.
60. Thomsen RW, Hundborg HH, Johnsen SP, Pedersen L, Sorensen HT, Schonheyder HC, et al. Statin use and mortality within 180 days after bacteremia: a population-based cohort study. Crit Care Med. 2006;34(4):1080-6.
61. Tragni E, Filippi A, Mazzaglia G, Sessa E, Cricelli C, Catapano AL. Monitoring statin safety in primary care. Pharmacoepidemiology and Drug Safety. 2007;16(6):652-7.
62. Wolozin B, Wang SW, Li NC, et al. Simvastatin is associated with a reduced incidence of dementia and Parkinson’s disease. BMC Med 2007;5:20. doi:10.1186/1741-7015-5-20.
63. Yaffe K, Barrett-Connor E, Lin F, Grady D, Yaffe K, Barrett-Connor E, et al. Serum lipoprotein levels, statin use, and cognitive function in older women. Archives of Neurology. 2002;59(3):378-84.

**Text S3. Newcastle-Ottawa Quality Assessment Scale**

**CASE CONTROL STUDIES**

Note: A study can be awarded a maximum of one star for each numbered item within the Selection and Exposure categories. A maximum of two stars can be given for Comparability.

**Selection**

1) Is the case definition adequate?

a) yes, with independent validation **🟑**

b) yes, eg record linkage or based on self reports

c) no description

2) Representativeness of the cases

a) consecutive or obviously representative series of cases **🟑**

b) potential for selection biases or not stated

3) Selection of Controls

a) community controls **🟑**

b) hospital controls

c) no description

4) Definition of Controls

a) no history of disease (endpoint) **🟑**

b) no description of source

**Comparability**

1) Comparability of cases and controls on the basis of the design or analysis

a) study controls for _______________ (Select the most important factor.) **🟑**

b) study controls for any additional factor **🟑** (This criteria could be modified to indicate specific control for a second important factor.)

**Exposure**

1) Ascertainment of exposure

a) secure record (eg surgical records) **🟑**

b) structured interview where blind to case/control status **🟑**

c) interview not blinded to case/control status

d) written self report or medical record only

e) no description

2) Same method of ascertainment for cases and controls

a) yes **🟑**

b) no

3) Non-Response rate

a) same rate for both groups **🟑**

b) non respondents described

c) rate different and no designation

**COHORT STUDIES**

Note: A study can be awarded a maximum of one star for each numbered item within the Selection and Outcome categories. A maximum of two stars can be given for Comparability

**Selection**

1) Representativeness of the exposed cohort

a) truly representative of the average _______________ (describe) in the community **🟑**

b) somewhat representative of the average ______________ in the community **🟑**

c) selected group of users eg nurses, volunteers

d) no description of the derivation of the cohort

2) Selection of the non exposed cohort

a) drawn from the same community as the exposed cohort **🟑**

b) drawn from a different source

c) no description of the derivation of the non exposed cohort

3) Ascertainment of exposure

a) secure record (eg surgical records) **🟑**

b) structured interview **🟑**

c) written self report

d) no description

4) Demonstration that outcome of interest was not present at start of study

a) yes **🟑**

b) no

**Comparability**

1) Comparability of cohorts on the basis of the design or analysis

a) study controls for _____________ (select the most important factor) **🟑**

b) study controls for any additional factor **🟑** (This criteria could be modified to indicate specific control for a second important factor.)

**Outcome**

1) Assessment of outcome

a) independent blind assessment **🟑**

b) record linkage **🟑**

c) self report

d) no description

2) Was follow-up long enough for outcomes to occur

a) yes (select an adequate follow up period for outcome of interest) **🟑**

b) no

3) Adequacy of follow up of cohorts

a) complete follow up - all subjects accounted for **🟑**

b) subjects lost to follow up unlikely to introduce bias - small number lost - > ____ % (select an adequate %) follow up, or description provided of those lost) **🟑**

c) follow up rate < ____% (select an adequate %) and no description of those lost

d) no statement

**Figure S3**

**Dementia (all)**

**Figure S4**

**Alzheimer’s disease**

**Figure S5**

**Dementia (no Alzheimer)**

**Figure S6**

**Parkinson’s disease**

**Figure S7**

**Peripheral Neuropathy**

**Figure S8**

**Depression**

**Figure S9**

**Age-related macular degeneration**

**Figure S10**

**Cataract**

**Figure S11**

**Glaucoma**

**Figure S12**

**Fractures**

**Figure S13**

**Venous thromboembolism**

**Figure S14**

**Myopathy**

**Figure S15**

**Liver disorders**

**Figure S16**

**Diabetes**

**Figure S17**

**Renal disorder**

**Figure S18**

**Rheumatoid arthritis**

**Figure S19**

**Pneumonia**
